# IMPROvER: the Integral Membrane Protein Stability Selector

**DOI:** 10.1038/s41598-020-71744-x

**Published:** 2020-09-16

**Authors:** Steven P. D. Harborne, Jannik Strauss, Jessica C. Boakes, Danielle L. Wright, James G. Henderson, Jacques Boivineau, Veli-Pekka Jaakola, Adrian Goldman

**Affiliations:** 1 Astbury Centre for Structural and Molecular Biology, University of Leeds, Leeds, UK; 2 Peak Proteins, BioHub, Alderley Park, Macclesfield, UK; 3 Novartis Institutes for Biomedical Research, Chemical Biology and Therapeutics, Virchow 16, Basel, Switzerland; 4 Confo Therapeutics, Technologiepark 94, 9052 Zwijnaarde, Belgium; 5 MIBS, Biological and Environmental Sciences, University of Helsinki, Helsinki, Finland

**Keywords:** Protein design, Structural biology, Software, Membrane proteins

## Abstract

Identifying stabilising variants of membrane protein targets is often required for structure determination. Our new computational pipeline, the Integral Membrane Protein Stability Selector (IMPROvER) provides a rational approach to variant selection by employing three independent approaches: *deep-sequence*, *model-based* and *data-driven*. In silico tests using known stability data, and in vitro tests using three membrane protein targets with 7, 11 and 16 transmembrane helices provided measures of success. In vitro, individual approaches alone all identified stabilising variants at a rate better than expected by random selection. Low numbers of overlapping predictions between approaches meant a greater success rate was achieved (fourfold better than random) when approaches were combined and selections restricted to the highest ranked sites. The mix of information IMPROvER uses can be extracted for any helical membrane protein. We have developed the first general-purpose tool for selecting stabilising variants of $\upalpha$-helical membrane proteins, increasing efficiency and reducing workload. IMPROvER can be accessed at http://improver.ddns.net/IMPROvER/.

## Introduction

Membrane proteins are key drug targets, but rational approaches to drug design against them have largely been hampered by a lack of high-resolution structures. Bottlenecks in solving membrane protein structures occur at every step, often because membrane proteins are unstable, especially in detergent^1^. The well-established scanning-mutagenesis approach has been highly successful at identifying stabilising variants. This brute-force trial-and-error method^2^ has led to improved expression, purification and ‘crystallisability’ of numerous membrane protein targets, often from the G-protein coupled receptor (GPCR) family (such as the $\upbeta _{1}$-adrenoceptor^2, 3^, adenosine $\hbox {A}_{{\mathrm{2A}}}$^4^ receptor and neurotensin receptor^5^). Two-thirds of all GPCR structures solved to date have had at least one stabilising amino acid change introduced. However, these scanning approaches are expensive and labour-intensive, which is a bottleneck for industry^6^ and intractable for most academic laboratories.

**Figure 1:**
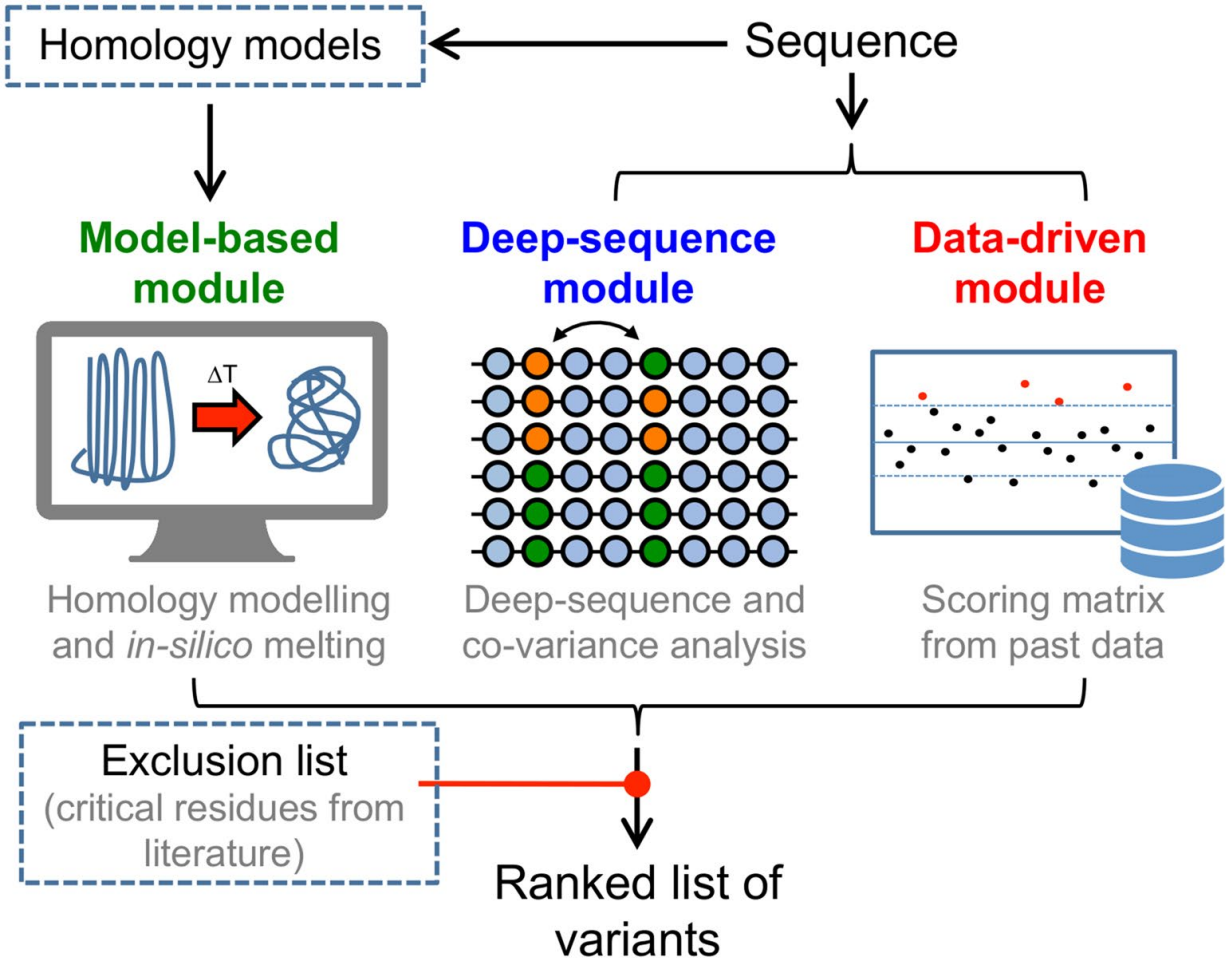
Graphical schematic for steps in the IMPROvER pipeline. Scheme of variant selection using IMPROvER, highlighting the three main modules: *deep-sequence* (blue), *model-based* (green) and *data-driven* (red). Once positions have been selected, they are compared against a list of residues to be excluded (e.g. those known to be critical to function from the literature).

Our solution was to create a computational tool that combines a number of bioinformatics techniques and prior data from membrane protein stabilisation studies to rank the likelihood of amino acid changes producing stabilising effects. Variants could then be prioritised based on rank. We anticipated that stabilising variants could be found with fewer experiments, increased speed and decreased cost and effort. Our new tool, the Integral Membrane Protein Stability Selector (IMPROvER), is a software package that takes sequence level information as input and produces a list of top-ranked stabilising variants (Fig. 1). It can also generate primer sequences required to make the variants by site-directed-mutagenesis. The methods used for selecting variants are based on the following three approaches: (1) *deep-sequence* analysis, (2) *model-based* analyses and (3) a *data-driven* analysis. Furthermore, we can use prior information about specific residues known to have a critical function in protein activity (or any other reason), and exclude these from selection (Fig. 1).

## Results

### Construction of IMPROvER modules

In the *deep-sequence* module of IMPROvER, highly redundant multiple sequence alignments ($>8,000$ sequences) were assembled to assess natural variation in protein sequence. Covariance^7^ between residues and natural frequency of residues at a position were used to identify potentially beneficial substitutions^8^. In this module, we used the EVcoupling and EVmutation open-source software^7, 8^.

In the model-based module, multiple input models were subjected to in silico saturation mutagenesis. A $\Delta \Delta$G of unfolding was rapidly calculated for each variant^9^. Sites with in silico improved thermostability in multiple models were ranked highest to help decrease bias from poorly modelled sites. In our tests, models were generated with comparative homology modelling where templates with correct fold were known (such as the GPCR test sets)^10^. Models for targets with unknown fold were generated using threading and ab-initio folding strategies^11^.

In the *data-driven* module, data from three GPCR stabilisation campaigns were analysed for both stabilising and destabilising trends. The GPCR dataset comprised of stability measurements for the majority of amino acid positions in turkey $\upbeta _{1}$ adrenergic receptor^12^, human adenosine $\hbox {A}_{{\mathrm{2A}}}$ receptor^13^ and rat neurotensin receptor 1^5^, when changed to alanine, or leucine if the residue at a position was natively alanine. Stability data for the $\hbox {A}_{{\mathrm{2A}}}$ receptor were available for apo, agonist and antagonist bound states; for neurotensin receptor 1 for the agonist and apo states; and for $\upbeta _{1}$ adrenergic receptor in the apo state only. On a position-by-position basis, both positive and negative effects on stability in GPCR stability datasets were correlated with bioinformatic information such as amino acid type, conservation, predicted topological position, predicted disordered region, predicted lipid-contact and predicted helix–helix contact scores. The bioinformatics information was calculated using several open-source packages including the PSIPRED suite^14^.

Whilst analysing the GPCR dataset, we observed that positions with amino acid conservation in the range of 50–60% were most associated with increased stability, while positions with amino acid conservation in the range of 80–90% were most associated with decreases in stability (Fig. S1; Table S1). This observation makes sense: highly conserved residues are less tolerant to substitution as they are in critical locations for fold or function so residue substitutions will tend to be detrimental. Conversely, poorly conserved sites tolerate substitution. Thus, substitutions at moderately conserved residues have a higher likelihood of increasing stability.

**Figure 2:**
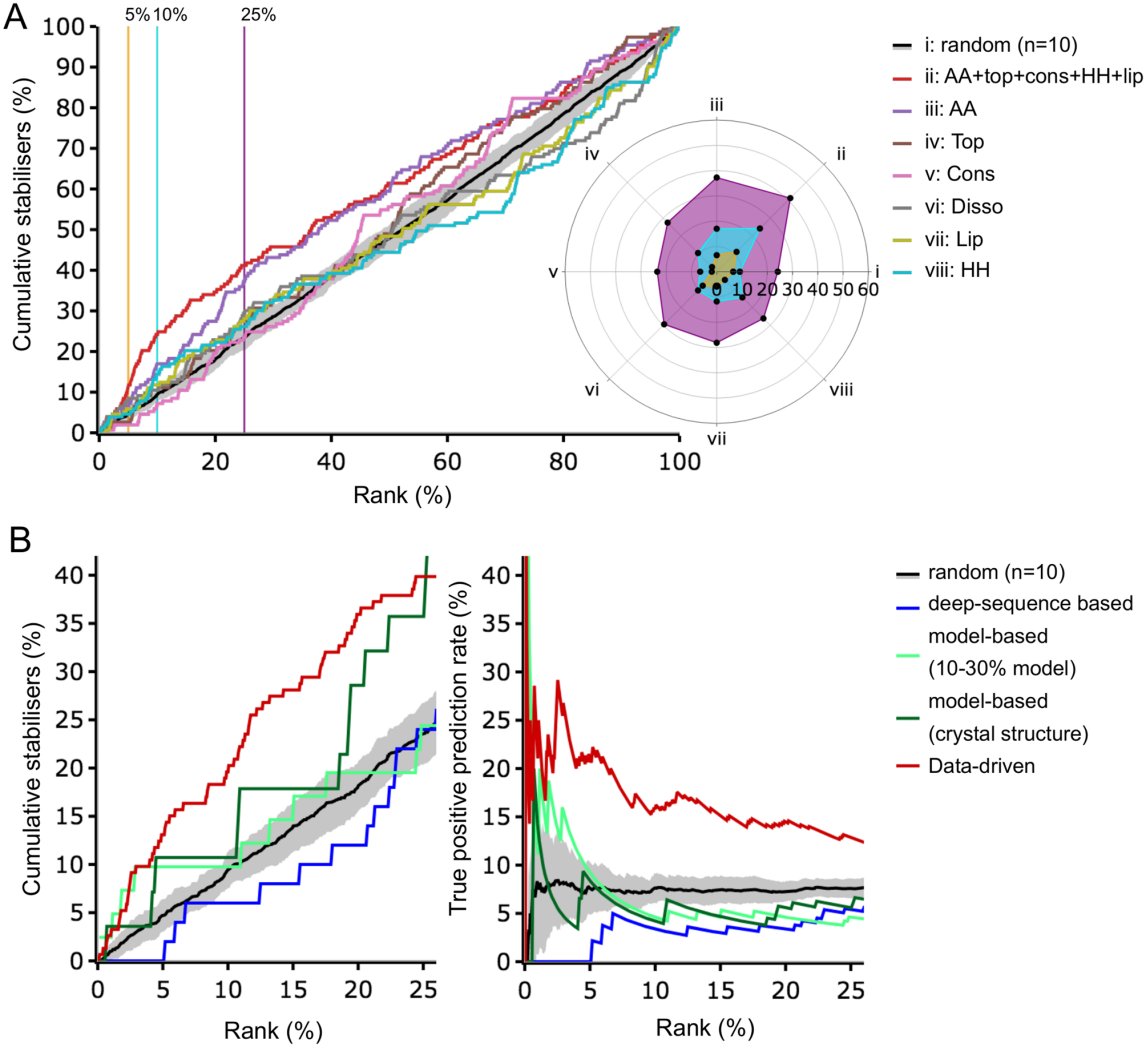
Performance of IMPROvER for predicting stability. (**A**) Cumulative scatter plot and radar plot to highlight the effect of different weightings of bioinformatic information in scoring methods to successfully group stabilising hits in the top 5, 10 or 25% of ranked lists (orange, cyan or purple in radar plot, respectively). Methods successful at selecting stabilising residues have lines above random (black) in scatter plot or points further outwards than random (i) in radar plot. Scoring methods tested: i. random, ii. combined score (for amino acid, topology, conservation, predicted helix–helix contact and predicted lipid contact) iii. amino acid score (AA), iv. topology score (Top), v. conservation score (Cons), vi. disorder score (Disso), vii. predicted lipid contact score (Lip) and viii. predicted helix contact score (HH). (**B**) GPCR stabilising hit data versus rank (position in list after sorting by score) from the three different IMPROvER modules: *deep-sequence* (blue), *model-based* using either homology model or crystal structure (light and dark green, respectively) and *data-driven* (red). Data are displayed with the Y-axis as a cumulative percent of stabilisers found (left) or true positive prediction rate (right). Data shown for random selection were the result of 10 random samplings of the dataset with the mean displayed (standard error is also indicated as grey shading).

In our analysis, we also found that substitution of residues G, T, A, Q, E and H were most associated with increases in stability (in descending order), and substitution of residues I, Y, W, D, C and V, were most associated with decreases in stability (in descending order) (Fig. S1). Similarly, when considering the topological location of residues, changes in transmembrane helices (TMH) most often stabilised, while changes in cytosolic helices most often destabilised. For the scoring method in this module, we found that amino acid identity, topological position and disorder prediction were the best predictors of effect on stability compared to amino acid conservation, which was a poorer indicator of stabilising effect (Fig. 2A). However, testing against the GPCR dataset showed that known stabilising hits were highly ranked when all the scoring methods apart from disorder prediction were combined (Fig. 2A). Therefore, this scoring method was the one used by the *data-driven* module for selecting variations to make.

### Testing IMPROvER against the GPCR dataset

The GPCR dataset also allowed us to evaluate the success of IMPROvER modules for correctly ranking stabilising and destabilising changes. For all methods, there was no strong correlation between stabilising rank (position after sorting by score) and the absolute thermostabilisation in the GPCR dataset. Instead, we considered the number of confirmed stabilising changes that were grouped at the top of ranked lists produced by each selection method (Fig. 2B). The *data-driven* approach appears to be the most successful, grouping approximately 40% (Fig. 2B) of the known stabilisers (Fig. 2B) in the first quartile. This observation is expected as the scoring method was derived from this set: we also tested the modules on naive datasets (see below).

The *model-based* method also grouped approximately 40% of known stabilisers in the first quartile, using crystal structures as input models (Fig. 2B). This result is also expected, as IMPROvER is intended as a tool that precedes crystal structure solution, and the quality of the model would be very high for homology models of known GPCR structures. We therefore prepared models from templates with 10–30% sequence identity with the targets in the GPCR dataset. Using these models, this approach performed well for the top 5–10% of ranked sites, but not well in the top quartile (Fig. 2B).

Sites in the first quartile selected by the *deep-sequence* approach were not better than expected from random selections (equal with the black line in Fig. 2B). However, only predictions of changes to alanine (or leucine if already alanine) were considered, as this matched the input GPCR dataset. The *deep-sequence* approach selects the best residue for each site from all 20 amino acids, not just alanine. Therefore, this approach may perform better given the freedom to select the best amino acid for each site. This hypothesis was explored when in vitro targets were tested (below). The model data suggest that individually each method will identify 25–40% of the stabilising variants for a new protein target if the first quartile of ranked residues is used. As stabilised proteins typically have less than ten point mutations in total, we were reasonably confident that our approach would allow us to identify a sufficient number of stabilising variants to stabilise membrane proteins of unknown structure.

### Stabilising variants of ClPPase selected with IMPROvER

**Figure 3:**
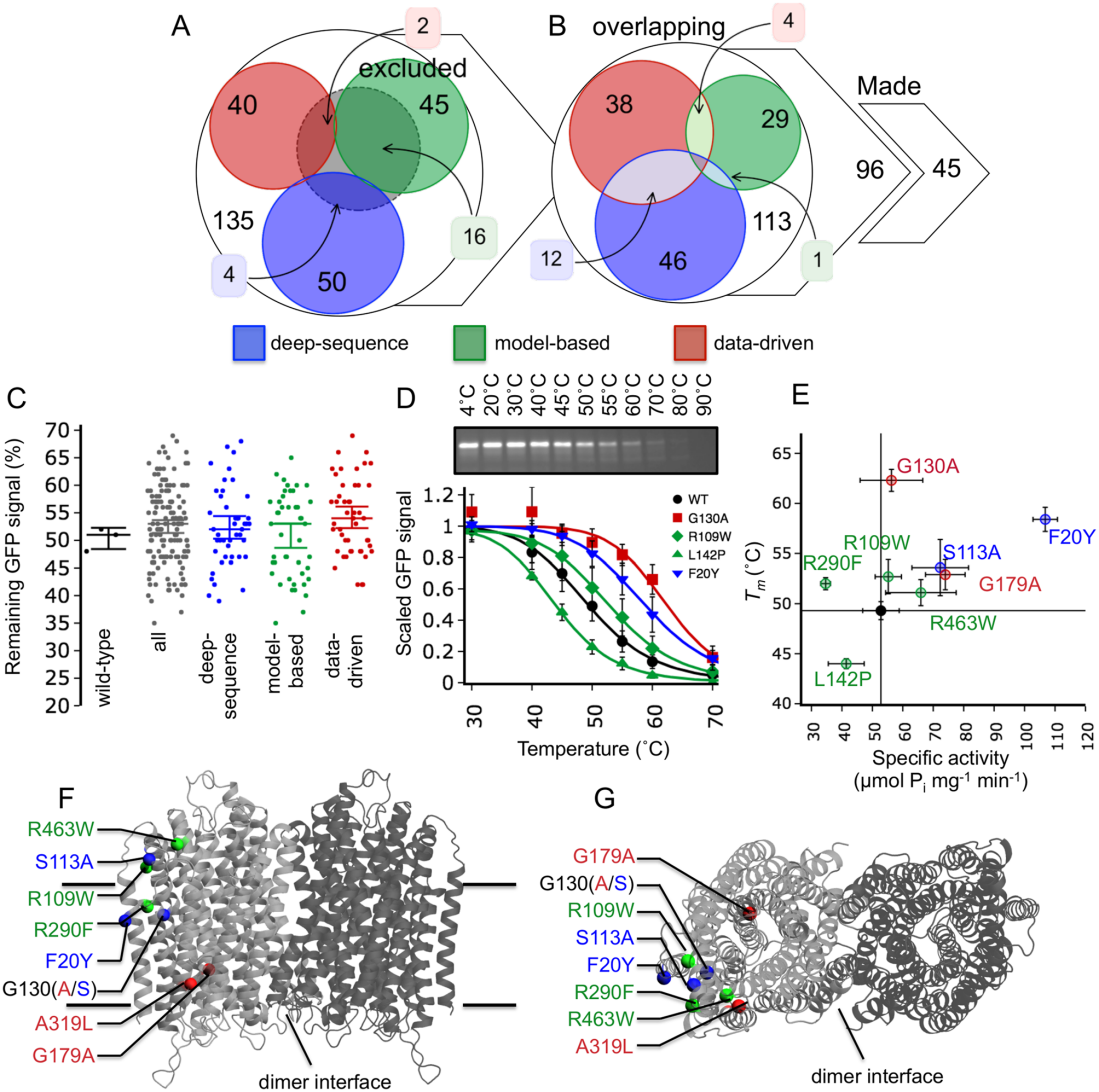
Using IMPROvER to identify stabilising variants of ClPPase. (**A**) Comparison of residues selected by IMPROvER modules but excluded due to prior known critical role. (**B**) Comparison of residues selected by IMPROvER that are overlapping between the different modules. (**C**) Statistical analysis of all data collected for ClPPase variant in-gel GFP-based stability assay, or grouped by prediction module. Each data point represents a biological repeat of the single-temperature stability analysis (single-temperature challenge at 50 $^{\circ }$C normalised to intensity of sample incubated on ice). In each case the median of all points is displayed with 95% confidence intervals represented as whiskers. (**D**) wild-type (WT) ClPPase, and representative variant curves of three stabilisers and one destabiliser, assayed by ten-temperature stability analysis (panel insert and Fig. S4) after temperature challenge at 20, 30, 40, 45, 50, 55, 60, 70, 80, and 90 $^{\circ }$C. Data in each curve are normalised to the intensity of sample incubated on ice. The wild-type curve was collected as an average of eight biological repeats and variants were three biological repeats. Please see Fig. S2A for an uncropped gel image. (**E**) $T_m$ of top stabilising variants (and the destabilising variant L142P) versus their specific $\hbox {PP}_{\mathrm{{i}}}$ hydrolysis activity. Error bars in all panels D and E are representative of SEM. Stabilising variants that retain or improve on wild-type activity cluster in the top right quadrant. (**F**) View in the plane of the membrane and (**G**) perpendicular to the membrane at the most stabilising variant positions (coloured balls) mapped to the best comparative homology model of ClPPase.

Our first in vitro test protein was a membrane-bound pyrophosphatase from *Clostridium leptum* (ClPPase) that was difficult to crystallise without further stabilisation. Scores given by IMPROvER were used to rank sites in the protein, with variants in the top 15% selected for stability testing (Table S2). 127 residues were specifically eliminated from testing as they were known to be critical in the mechanism of membrane-bound pyrophosphatases (Table S3)^15–26^, thus excluding 22 high-ranking variants (Fig. 3A). The sites selected by the different approaches were mostly non-overlapping. In 17 cases, sites were highly ranked by two different approaches, although the amino acid substitution was usually different (Fig. 3B). For the majority of overlapping cases, we selected the variant with the highest IMPROvER score and excluded the others, even if the amino acid change was different. 45 mutations were successfully introduced into the ClPPase gene by site-directed mutagenesis, providing a random sampling of half the sites ranked in the top 15% by IMPROvER: 15 from *deep-sequencing*, 15 from *model-based* and 15 from *data-driven* modules.

Expression of the variants in yeast, membrane isolation and solubilisation in dodecyl maltoside (DDM) allowed us to assay the thermostability of these variants in crude extracts using green fluorescent protein (GFP) fluorescence. Stability testing was based on heating the sample to a defined temperature, removing heat-precipitated protein by centrifugation and assessing remaining protein in solution by in-gel fluorescence. Using a ten-temperature melting analysis based on this method, the apparent melting temperature ($T_m$) determined for ClPPase was $49.3 \pm 0.9$ $^{\circ }$C (Fig. 3D and Fig. S2). To facilitate rapid testing of variants, they were subjected to a challenge at a single temperature and the remaining GFP-signal compared to that of wild-type (Fig. S2). 14 of 45 variants were identified as potentially stabilising. This selection was based on variants retaining over 55% of the wild-type GFP-signal, a cut-off determined by calculating the 95% confidence interval of all measurements in the dataset (Fig. 3C and Fig. S3).

To confirm initial hits as stabilising and gain an understanding of how successful the single-temperature assay was for identifying hits, we determined the $T_m$ for 21 of our variants using ten-temperature melting analyses (Table 1; Fig. 3D and Fig. S4). These 21 comprised 14 identified as stabilisers, 2 destabilisers and 5 neutral from the single-temperature assays. Only variants increasing the $T_m$ by $\ge$1.3 $^{\circ }$C (average SEM of $\Delta T_m$) compared to wild-type were considered stabilising in the ten-temperature assay. With data from the ten-temperature assay factored in, the overall success rate for selecting stabilising variants of ClPPase was 27%, which corresponds to 12 stabilising variants found. In contrast, we only found 2 destabilising variants (4% rate).

**Table 1:** Results from ten-temperature melting analysis of ClPPase variants.

Module	Variant	$T_m$ ($^{\circ }$C)$^{\mathrm{a}}$	$T_m$ error ($\pm ^{\circ }$C)$^{\mathrm{b}}$	$\Delta T_m$ ($^{\circ }$C)	$\Delta T_m$ error ($\pm ^{\circ }$C)$^{\mathrm{c}}$	Repeats (n)	Status
n/a	Wild-type	49.3	0.9	0.0	1.2	8	n/a
Deep-sequence	S113A	53.6	2.8	4.3	3.0	3	Stabilising
Deep-sequence	S273V	48.8	–	− 0.5	–	1	Neutral
Deep-sequence	G130S	57.4	–	8.1	–	1	Stabilising
Deep-sequence	S371K	50.6	–	1.3	–	1	Neutral
Deep-sequence	F20Y	58.4	1.2	9.1	1.5	3	Stabilising
Model-based	V81W	50.5	–	1.2	–	1	Stabilising
Model-based	R463W	51.1	1.3	1.8	1.6	3	Neutral
Model-based	L142P	44.0	0.5	− 5.3	1.0	3	Destabilising
Model-based	L151I	49.7	1.2	0.4	1.5	2	Neutral
Model-based	V693Y	51.4	–	2.1	–	1	Stabilising
Model-based	R109W	52.7	1.7	3.4	1.9	3	Stabilising
Model-based	R290F	50.5	–	1.2	–	3	Stabilising
Model-based	D468F	52.0	0.6	2.7	1.1	1	Stabilising
Model-based	I501L	50.6	–	1.3	–	1	Neutral
Model-based	G179A	52.9	1.5	3.6	1.7	3	Stabilising
Data-driven	A492L	47.7	–	− 1.6	–	1	Destabilising
Data-driven	A14L	48.4	–	− 0.9	–	1	Neutral
Data-driven	G31A	51.0	–	1.7	–	1	Stabilising
Data-driven	A319L	51.5	–	2.2	–	1	Stabilising
Data-driven	G130A	62.3	1.1	13.0	1.4	3	Stabilising
Data-driven	A114L	50.2	–	0.9	–	1	Neutral
Combined	G130A+F20Y	59.6	1.8	10.3	2.0	3	Stabilising

$^{\mathrm{a}}$Average $T_m$ was calculated from individual $T_m$ estimated for each individual repeat by fitting with a four-parameter dose-response curve (variable slope) by non-linear least-squares fitting in the python package *scipy.stats*.

$^{\mathrm{b}}$For n > 1: standard error of the mean (SEM) shown.

$^{\mathrm{c}}$For n > 1: error calculated and propagated as detailed in “Methods and materials” section.

Variant G130A had the biggest increase in $T_m$ of 13.0 ± 1.4 $^{\circ }$C, followed by F20Y and G130S with increases of 9.1 ± 1.5 $^{\circ }$C and 8.1 $^{\circ }$C, respectively (Table 1; Fig. 3D,E and Fig. S4). The false-positive and false-negative rates of the single-temperature assay were both 15% (Fig. S3). The different approaches were similarly powerful at ranking variants with success rates of 20%, 33% and 27%, for *deep-sequence*, *model-based* and *data-driven*, respectively (Fig. 3C and Fig. S3). These success rates are 3–4 times greater than expected by random selection (7.6% in GPCRs) and similar to the in silico testing of the GPCR dataset (Fig. 2B). Furthermore, all top stabilising hits retained or improved upon wild-type $\hbox {PP}_{\mathrm{{i}}}$ hydrolysis activity (Fig. 3E and Fig. S3C).

Understanding why certain amino acid substitutions stabilise ClPPase is difficult without an experimental three-dimensional structure. However, if the positions of the variants are mapped to our best comparative homology model, all stabilising variants cluster on the opposite side of the protein from the dimer interface, close to regions that interact with lipid (Fig. 3F,G). Furthermore, 6 of the 12 stabilising variants (G130A, G130S, R290F, F20Y, S113A and R109W) cluster at the interface between helices 1, 3, 4 and 7 (Fig. S5). This region includes the variants that stabilise ClPPase the most. We have previously shown that this part of the protein is particularly mobile in molecular dynamics simulations using the *Thermotoga maritima* homologue^27^.

It is possible that these six variants act to stabilise helix–helix interaction, particularly around TMH1. G130 (15% conserved) stabilises ClPPase when substituted to serine or alanine, possibly constraining movement of TMH4 relative to TMH1, by replacing the helix-breaking glycine residue (Fig. S5A,B). F20 (10% conserved) on TMH1 stabilised when substituted to tyrosine, possibly by providing hydrogen bonding to TMH7 R290 (34% conserved), increasing the rigidity of this region (Fig. S5C). Interestingly, the R290F substitution also increases stability, possibly due to energy reduction on removing repulsion between R290 and R24, by increasing hydrophobic packing between TMH4 and TMH7, or both (Fig. S5D). Both S113 (2% conserved) and R109 (96% conserved) stabilise ClPPase when substituted with alanine or tryptophan, respectively, and are at the C-terminus of TMH3 in an interface between TMH3 and TMH1. It is likely they stabilise the interaction between these helices by improving the hydrophobic packing in this region (Fig. S5E,F). Therefore, it appears that all these substitutions act to stabilise through a similar mechanism: increasing rigidity in the helix–helix interfaces between TMHs 1, 3, 4 and 7. This may explain why combining these variants together did not additively increase protein stability as seen in the G130A/F20Y combined variant (Fig. S3C).

### Stabilising variants of hENT1 selected with IMPROvER

**Figure 4:**
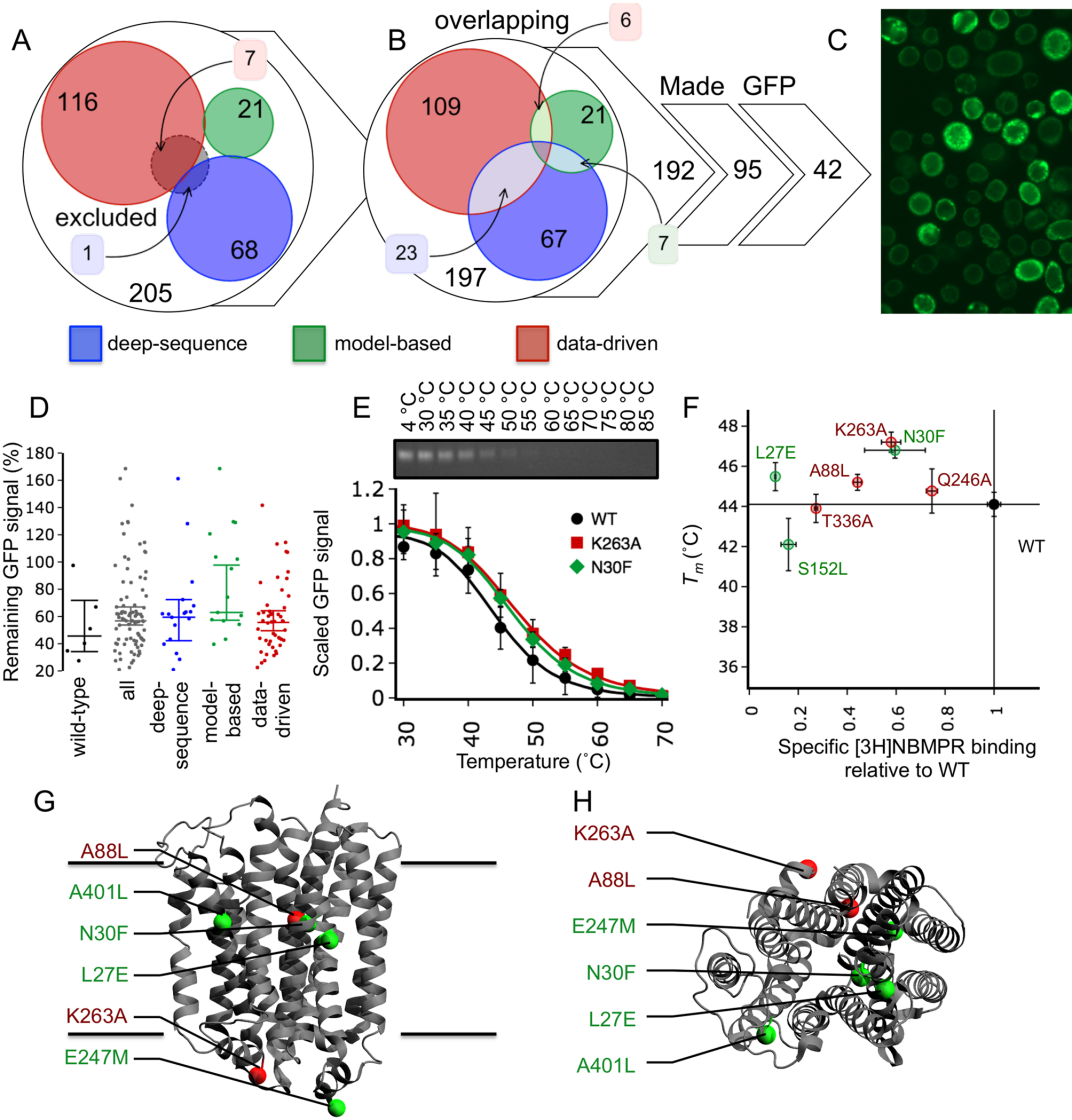
Using IMPROvER to identify stabilising variants of hENT1. (**A**) Assessment of residues selected as sites for variation by multiple modules in the IMPROvER pipeline. (**B**) Comparison of which residues were predicted by IMPROvER modules but excluded due to prior known critical role. (**C**) Representative example of the GFP-fluorescence observed in *Sf9* insect cells expressing GFP-tagged hENT1 variants (cells were expressing variant E264A). (**D**) Statistical analysis of data collected for hENT1 variant in-gel GFP-based stability assay together, wild-type alone or separated by the module that selected it. Each data point represents a biological repeat of the assessment of protein surviving after being challenged with a temperature of 50 $^{\circ }$C compared to 4 $^{\circ }$C sample. In each case the median of all points is displayed with 95% confidence intervals represented as whiskers. (**E**) Wild-type (WT) hENT1 stability assayed by in-gel GFP analysis (panel insert) after temperature challenges at 30, 45, 40, 45, 50, 55, 60, 65, 70, 75, 80 and 85 $^{\circ }$C. Wild-type and curves were collected as an average of 12 repeats, whereas variant curves were collected as an average of 2–5 repeats. Double banding pattern on SDS-PAGE attributed to glycosylation of N48^28^. The sum of intensities for both bands is used as a marker of stability. Data in each curve are normalised to the intensity of sample incubated on ice. Please see Fig. S7A for an uncropped gel image. (**F**) Scatter plot of hENT1 variant stability versus amount of radiolabelled specific inhibitor [3*H*]-NBMPR bound in the membrane relative to wild-type. Error bars in panels (**E**) and (**F**) are representative of SEM. (**G**) View in the plane of the membrane and **H**) perpendicular to the membrane at most stabilising variant hits mapped to the crystal structure of dilazep bound hENT1 (PDB: 6OB7)^40^. Residues E247 and K263 were absent from hENT1 crystal structures, and so the closest residue visible to the missing one has been used (residues 240 and 281, respectively).

Our second in vitro test protein was the human equilibrative nucleoside transporter isoform 1 (hENT1). As with ClPPase, IMPROvER was used to rank variants for stability testing (Table S4). 36 positions were excluded based on the literature (Table S5)^28–39^, thus eliminating eight high-ranking variants (Fig. 4A). A larger number of 36 hENT1 residues were highly ranked by more than one approach compared to ClPPase, possibly because hENT1 is much shorter, and in total more residues were selected for study (Fig. 4B). Unlike for ClPPase, we excluded overlapping variants only if the amino acid substitution was the same (5 cases). 95 mutations were successfully introduced into the hENT1 gene by site-directed mutagenesis, providing a random sampling of half the sites ranked in the top 40% by IMPROvER. However, due to the extra technical requirements of Bacmid formation during expression in insect cells, only 75 viruses were generated. Of these, 41 displayed GFP-fluorescence localised at the cell membrane (Fig. 4C and Fig. S6), bringing the tally down to a random sampling of a fifth of the sites ranked in the top 40%: 10 *deep-sequence*, 7 *model-based* and 24 *data-driven*.

hENT1 variants were expressed in insect cells, solubilised in DDM and single-temperature stability assays carried out using the GFP-signal relative to that of the wild-type. 12 out of 41 variants tested were classified for further stability testing, an initial success rate of 29% (Figs. S7, S8A). This selection of stabilising hENT1 variants was based on retention of over 63% of the wild-type GFP-signal, a cut-off determined by calculating the 95% confidence interval of all measurements in the dataset (Fig. 4D).

The apparent $T_m$ determined for wild-type hENT1 was 44.6 ± 0.6 $^{\circ }$C (Table 2; Fig. 4E and Fig. S9), using the ten-temperature stability assay. Using results from the single-temperature assay for classification, all apparent stabilising, several neutral and several destabilising variants were tested using the ten-temperature assay. Increases in hENT1 stability were modest compared to those observed for ClPPase, with the effects being 2.6 ± 1.3 $^{\circ }$C (K263A), 2.2 ± 0.7 $^{\circ }$C (N30F) and 1.2 ± 0.8 $^{\circ }$C (E247M) (Table 2; Fig. 4E, Figs. S8B, S9). Using a 0.9 $^{\circ }$C cut-off (average SEM $\Delta T_m$), five hENT1 variants were considered to be stabilising (Table 2). With the relaxation of selection to the top 40% of sites and the sparsity of coverage, IMPROvER was less successful at predicting stabilisation than for the ClPPase case (12%). However, if only the top 10% of ranked sites are considered that rate increases to 16%, over double that expected for random selections (7.6% in GPCRs). The *model-based* approach was most successful with a rate of 43%, followed by *data-driven* at 8% (Fig. 4E and Fig. S9). Of the variants suggested by the *deep-sequence* module, none we tested were stabilising. We found 15 destabilising hENT1 variants, more than ClPPase, but at a 36% rate, this was still lower than expected for random selections (48.5% in GPCRs).

**Table 2:** Results from ten-temperature melting analysis of hENT1 variants.

Module	Variant	$T_m$ ($^{\circ }$C)$^{\mathrm{a}}$	$T_m$ error ($\pm ^{\circ }$C)$^{\mathrm{b}}$	$\Delta T_m$ ($^{\circ }$C)	$\Delta T_m$ error ($\pm ^{\circ }$C)$^{\mathrm{c}}$	Repeats (n)	Status
n/a	Wild-type	44.6	0.7	0.0	0.9	12	n/a
Deep-sequence	I380V	42.0	0.1	− 2.5	0.6	2	Destabilisng
Deep-sequence	M306T	41.3	0.1	0.4	1.8	2	Neutral
Deep-sequence	G225V	42.1	0.1	− 1.0	0.9	2	Destabilisng
Deep-sequence	S321T	44.9	1.7	− 2.5	0.6	2	Destabilisng
Model-based	R233L	37.1	1.6	− 3.8	0.6	2	Destabilisng
Model-based	S152L	42.7	1.3	− 1.9	1.4	5	Destabilisng
Model-based	E247M	45.8	0.6	1.2	0.8	4	Stabilising
Model-based	N30F	46.8	0.4	2.2	0.8	5	Stabilising
Model-based	L27E	45.5	0.7	0.9	0.9	5	Stabilising
Data-driven	A401L	46.3	0.7	1.7	1.0	2	Stabilising
Data-driven	T336A	43.9	0.7	− 0.7	0.9	5	Neutral
Data-driven	Q246A	47.6	1.1	0.2	0.9	5	Neutral
Data-driven	F153A	40.8	0.1	− 7.5	1.7	2	Destabilisng
Data-driven	G207A	43.6	0.7	− 3.3	0.6	2	Destabilisng
Data-driven	A88L	45.2	0.7	0.8	0.9	5	Neutral
Data-driven	K263A	47.2	1.1	2.6	1.3	5	Stabilising

$^{\mathrm{a}}$Average $T_m$ was calculated from individual $T_m$ estimated for each individual repeat by fitting with a four-parameter dose-response curve (variable slope) by non-linear least-squares fitting in the python package *scipy.stats*.

$^{\mathrm{b}}$ For n > 1: standard error of the mean (SEM) shown.

$^{\mathrm{c}}$ For n > 1: error calculated and propagated as detailed in “Methods and materials” section.

As with ClPPase, we wanted to rationalise why the amino acid substitutions identified were able to stabilise hENT1. When we started this work, no crystal structure of hENT1 was available. Recently, the structure of hENT1 has been solved^40^, which allows us to map the positions of successful stabilising variants to the structure (Fig. 4G,H). We observed a cluster of N30 (83% conserved) and L27 (56% conserved) on TMH1, which stabilised hENT1 when changed to phenylalanine or glutamate, respectively (Fig. S10A). These substitutions are likely to stabilise TMH1 by improving packing between it and adjacent helices. It should be noted that the L27E variant abolished NBMPR binding, whereas N30F did not (Fig. 4F and Fig. S8C). Although L27 is close to the NBMPR binding site, the side-chain is on the opposite side of the helix facing the protein-lipid interface, not into the binding site (Fig. S10A). The second cluster of sites included E247 (18% conserved) and K263 (2% conserved), which stabilised hENT1 when substituted to methionine or alanine, respectively. These sites are located in the intracellular domain between helices 6 and 7. This entire region (residue 243–274) was deleted from the crystallographic constructs^40^, and is likely to be unstructured. The E247M or K263A variants presumably stabilise the protein by reducing conformational flexibility in this region. Although we did not test it in the ten-temperature stability assay, it is notable that E265A that was indicated to be stabilising by the single-temperature assay is also part of this loop.

### Stabilising variants of $\hbox {hPTH}_{1}\hbox {R}$ selected with IMPROvER

**Figure 5:**
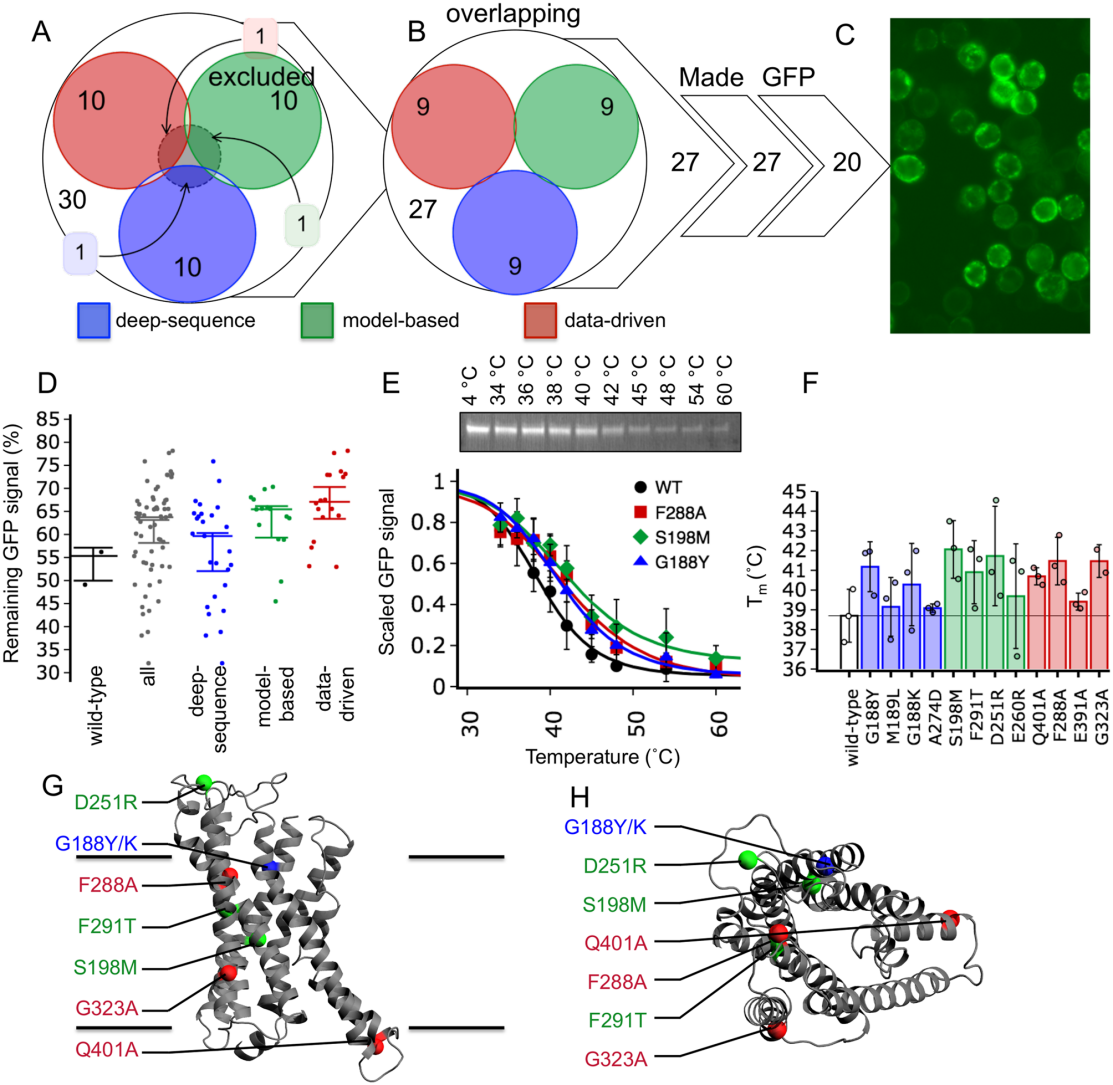
Using IMPROvER to identify stabilising variants of $\hbox {hPTH}_{1}\hbox {R}$. (**A**) Assessment of residues selected as sites for variation by multiple modules in the IMPROvER pipeline. (**B**) Comparison of which residues were predicted by IMPROvER modules but excluded due to prior known critical role. (**C**) Representative example of the GFP-fluorescence observed in *Sf9* insect cells expressing GFP-tagged $\hbox {hPTH}_{1}\hbox {R}$ variants (cells were expressing variant Q401A). (**D**) Statistical analysis of data collected for $\hbox {hPTH}_{1}\hbox {R}$ variant in-gel GFP-based stability assay together, wild-type alone or separated by the module that selected it. Each data point represents a biological repeat of the assessment of protein surviving after being challenged with a temperature of 39 $^{\circ }$C compared to 4 $^{\circ }$C sample. In each case the median of all points is displayed with 95% confidence intervals represented as whiskers. (**E**) Wild-type (WT) $\hbox {hPTH}_{1}\hbox {R}$ stability assayed by in-gel GFP analysis (panel insert) after temperature challenges at 4, 34, 36, 38, 40, 42, 45, 54, and 60 $^{\circ }$C. Wild-type and variant curves were collected as an average of three repeats. Data in each curve are normalised to the intensity of sample incubated on ice. Please see Fig. S11A for an uncropped gel image. (**F**) Barchart of $\hbox {hPTH}_{1}\hbox {R}$ variant $T_m$ relative to wild-type. Error bars in panels (**E**) and (**F**) are representative of SEM. (**G**) View in the plane of the membrane and (**H**) perpendicular to the membrane at most stabilising variant hits mapped to the crystal structure of $\hbox {hPTH}_{1}\hbox {R}$ (PDB: 6FJ3)^47^.

Our third in vitro test protein was the human parathyroid hormone receptor isoform 1 ($\hbox {hPTH}_{1}\hbox {R}$) a family B GPCR. A $\hbox {hPTH}_{1}\hbox {R}$ construct in which the N-terminal extracellular domain had been replaced with a BRIL fusion was used for the introduction of stabilising variants. Only the top 10% of sites suggested by IMPROvER were considered for introduction into $\hbox {hPTH}_{1}\hbox {R}$ (Table S6). 32 positions were excluded based on the literature (Table S7)^41–46^, thus eliminating three out of the 30 highest-ranking variants (Fig. 5A). None of the remaining sites were overlapping predictions between different IMPROvER modules (Fig. 5B). However, unlike ClPPase and hENT1, different variations at the same site were allowed if highly ranked, which occurred in two cases (G188 and T427). 20 of the remaining variants were successfully introduced by site-directed mutagenesis, and showed clear GFP-fluorescence when expressed in *Sf9* insect cells (Fig. 5C): 9 from *deep-sequencing*, 5 from *model-based* and 5 from *data-driven* modules.

The apparent $$T_m$$ determined for wild-type $$\hbox {BRIL-PTH}_{1}\hbox {R}$$ solubilised in DDM was 38.7 ± 0.8 $$^{\circ }$$C (Fig. 5E and Fig. S11). Therefore, single-point stability assays were performed at 39 $$^{\circ }$$C for all variants, and GFP-signal relative to that of the wild-type analysed. 10 out of 20 variants were potentially stabilising as they retained over 63% of the wild-type GFP-signal, a cut-off determined by calculating the 95% confidence interval of all measurements in the dataset (Fig. 5D). These ten variants and two borderline cases were tested using the ten-temperature stability assay, confirming eight variants of $$\hbox {PTH}_{1}\hbox {R}$$ with an increase in $$T_m$$ of $$>1.1$$$$^{\circ }$$C (average SEM of $$\Delta T_m$$), an overall success rate of 40% (Table 3, Table S6; Fig. S11). The variant S198M had the biggest increase in stability with a 3.4 ± 1.1 $$^{\circ }$$C increase in $$T_m$$, followed by F288A, G323A and G188Y (2.8 ± 0.7 $$^{\circ }$$C, 2.8 ± 0.6 $$^{\circ }$$C and 2.5 ± 0.7 $$^{\circ }$$C, respectively). These increases in stability are relatively modest in comparison to those observed for ClPPase, but similar to hENT1. The *model-based* and *data-driven* approaches performed best with success rates of 60% and 50%, respectively. The *deep-sequence* approach was less successful for $$\hbox {hPTH}_{1}\hbox {R}$$ with a success rate of 22%. None of the $$\hbox {PTH}_{1}\hbox {R}$$ variants tested in ten-temperature stability assays were destabilising and only one variant had a significantly decreased survival in single-temperature assay, a very low rate of 5% destabilisers selected.

**Table 3:** Results from ten-temperature melting analysis of $\hbox {hPTH}_{1}\hbox {R}$ variants.

Module	Variant	$T_m$ ($^{\circ }$C)$^{\mathrm{a}}$	$T_m$ error ($\pm ^{\circ }$C)$^{\mathrm{b}}$	$\Delta T_m$ ($^{\circ }$C)	$\Delta T_m$ error ($\pm ^{\circ }$C)$^{\mathrm{c}}$	Repeats (n)	Status
n/a	Wild-type	38.7	0.8	0	1.1	3	n/a
Deep-sequence	G188Y	41.2	0.7	2.5	0.7	3	Stabilising
Deep-sequence	M189L	39.1	0.9	0.4	0.9	3	Neutral
Deep-sequence	G188K	40.3	1.2	1.6	1.2	3	Stabilising
Deep-sequence	A274D	39.1	0.1	0.4	0.1	3	Neutral
Model-based	S198M	42.1	0.8	3.4	1.1	3	Stabilising
Model-based	F291T	40.9	0.9	2.2	1.2	3	Stabilising
Model-based	D251R	41.7	1.5	3.0	1.6	3	Stabilising
Model-based	E260R	39.7	1.5	1.0	1.7	3	Neutral
Data-driven	Q401A	40.7	0.2	2.0	0.2	3	Stabilising
Data-driven	F288A	41.5	0.7	2.8	0.7	3	Stabilising
Data-driven	E391A	39.4	0.2	0.7	0.2	3	Neutral
Data-driven	G323A	41.5	0.6	2.8	0.6	2	Stabilising

$^{\mathrm{a}}$Average $T_m$ was calculated from individual $T_m$ estimated for each individual repeat by fitting with a four-parameter dose-response curve (variable slope) by non-linear least-squares fitting in the python package *scipy.stats*.

$^{\mathrm{b}}$For n > 1: standard error of the mean (SEM) shown.

$^{\mathrm{c}}$For n > 1: error calculated and propagated as detailed in “Methods and materials” section.

Rationalisation of the stabilising $\hbox {PTH}_{1}\hbox {R}$ variants was possible by mapping their positions to the recently solved $\hbox {PTH}_{1}\hbox {R}$ crystal structure (PDB: 6FJ3; Fig. 5G,H)^47^. As with ClPPase and hENT1, we observed clustering of certain stabilising variants. For example, F288 (35% conserved) and F291 (38% conserved) are one helix turn apart from one another on TMH3, and are involved in the helix–helix interaction between TMH2 and TMH3 (Fig. S10B). Mutation to alanine or threonine, respectively, likely increases stability by improving the helix–helix packing interaction between TMH2 and TMH3. Furthermore, G188 (1% conserved) and S198 (89% conserved) are both on TMH1, with G188 facing into a void between TMH1 and TMH2 and S198 involved in the helix–helix interaction between TMH1 and TMH7 (Fig. S10C). Notably, the position G188 has previously been shown to assist $\hbox {PTH}_{1}\hbox {R}$ stability, and was mutated to alanine in the active state structure of $\hbox {PTH}_{1}\hbox {R}$ solved by cryo-EM^48^. Here, both G188Y and G188K variants were tested, both drastic changes that add bulky charged or hydrophilic groups, and both changes increased stability. It is likely that variation at G188 and S198 increases stability in $\hbox {PTH}_{1}\hbox {R}$ by increasing rigidity in TMH1 by improving helix–helix packing on either side.

## Discussion

Numerous methods have been used to identify stabilising variants of membrane proteins, with scanning-mutagenesis being arguably the most successful. Directed evolution by random mutagenesis has also shown some success^49, 50^. These in vitro methods, although successful, are time and resource consuming, or suffer from biases that restrict the variations possible or the type of targets that can be worked on^51^. Here, we describe a new computational tool called IMPROvER, the first general-purpose tool to rapidly select stabilising variants of multi-pass $\upalpha$-helical membrane proteins in silico for structural and biophysical characterisation.

### Methodology, performance and use of the IMPROvER software

All three modules identified stabilising variants better than random (Fig. 2B). Predictions from the different modules were mostly non-overlapping, as they interrogated protein sequence space differently. Thus, the predictive power was additive, allowing a success rate of as much as fourfold above random for ClPPase and $\hbox {hPTH}_{1}\hbox {R}$.

We specifically chose a broad range of top-ranked residues to calibrate the correlation between calculated rank position and stabilisation: for hENT1 in the top 40%, for ClPPase in the top 15% and $\hbox {hPTH}_{1}\hbox {R}$ the top 10%. Overall, the IMPROvER rank scores do not correlate directly with the experimentally determined $\Delta T_m$ (Tables S2 and S4) and thus do not provide exact predictions of stability. Rather, they guide a rational approach to selecting sites, and the sites found by IMPROvER and those used for crystallography^40^ are correlated. For instance, both E247M and K263A are in the loop deleted in the hENT1 crystal structure, and N288K, ranked 10th by IMPROvER, was used in the structure solution of hENT1. We nonetheless had much higher success rates for $\hbox {hPTH}_{1}\hbox {R}$ and ClPPase than hENT1, likely due to the more stringent selection criteria.

For researchers wishing to use IMPROvER on their membrane protein targets, we suggest equal selection of predictions from each of the IMPROvER modules. Furthermore, those selections should be in the top 5–10% of predicted sites. For example, the top ten ranked selections should be taken from each of the IMPROvER modules for a membrane protein of 300 amino acids to provide 30 sites total as we did for $\hbox {hPTH}_{1}\hbox {R}$. We anticipate success rates of 25–40% in line with our test cases if this method is followed.

### Identification and use of non-additive effects

Stabilising variants clustered together in three-dimensional space in protein structure in ClPPase, hENT1 and $\hbox {hPTH}_{1}\hbox {R}$ (Figs. 3F, 4G, 5G); certain parts of the proteins, such as the 246–284 loop in hENT1, seem particularly unstable. Stabilising variations can have non-additive effects, such as two negative charges that interact, where changing one half may impart much or all of the effect of changing both. This has been observed in GPCRs^2, 6^, and appears to be true when trying to combine the two most stabilising variants of ClPPase (F20Y and G130A). A rational approach to combining stabilising variants together is important, and we have previously implemented the powerful statistical method of fractional factorial analysis for such a combination of effects in membrane proteins^52^. Our current version of IMPROvER is thus the first step in our pipeline for efficient protein stabilisation engineering.

In GPCRs, a number of ‘hot-spots’ are already known where specific variation often confers stability. Such data are not yet available for other membrane protein families but the data generated by using IMPROvER will begin to fill these gaps in our understanding. The TMH 1/3/4/7 interface in membrane-bound PPases, and the two clusters identified in nucleoside transporters, may be such ‘hot-spots’.

### Comparison of IMPROvER to other computational methods

A number of the other in silico methods for selecting stabilising variants are also available. For example, the use of deep-sequence analysis combined with organism growth temperature to guide the introduction of thermophile specific amino acid adaptations into mesophilic homologues was successful for the stabilisation of tetracycline transporter (TetL) from *Bacillus subtilis*^53^. The major drawback of this approach is the requirement for thermophilic versions to exist in nature, which is not always true for higher eukaryotic targets.

There have also been a number of other in silico approaches, mainly focussing on selecting stabilising variants of GPCRs. For example, the use of molecular dynamics combined with statistical thermodynamics successfully predicted two highly stabilising variants of $\hbox {A}_{{\mathrm{2A}}}\hbox {R}$ prior to experimental confirmation^54^. This method took advantage of existing crystal structures of $\hbox {A}_{{\mathrm{2A}}}\hbox {R}$ to perform calculations, thus, would not be appropriate for novel targets. Similarly, methods based on ligand induced transmembrane conformational changes (LiTiCOn) in GPCRs were used to create conformational ensembles of models for a number of GPCRs. Those models were then used for in silico identification of stabilising variants that could be confirmed from prior experimental information^55^.

More recently, machine-learning approaches have been applied, in which the existing GPCR dataset of stabilising variants was used as the training set to successfully identify stabilising variants in naive sequences^56^. One piece of software that has used this approach is the software CompoMug (Computational Predictions of Mutations in GPCRs)^57^, which showed a 25% success rate for the identification of stabilising 5-$\hbox {HT}_{\mathrm{2C}}$ variants for crystallographic study. Furthermore, CompoMug also uses a combined approach similar to IMPROvER, with modules equivalent to the *deep-sequence*, *model-based* and *data-driven* IMPROvER modules used in combination^57^. However, the underlying software used for calculations in each of these modules is not the same between CompoMug and IMPROvER. Importantly, we have validated that IMPROvER works across structurally distinct $\upalpha$-helical membrane protein targets not just GPCRs, and our success rates are comparable or better than these other pieces of software.

### Conclusions and future outlook

There will always be a need for the stabilisation of recalcitrant membrane protein targets, like ClPPase, hENT1 and $\hbox {hPTH}_{1}\hbox {R}$, and using a combination of tools is often necessary for success. These targets are important not only for high-resolution structure determination, but also other biophysical techniques, including but not limited to, surface plasmon resonance, isothermal titration calorimetry and microscale thermophoresis.

The dataset used for constructing the scoring matrix in the *data-driven* approach is currently limited as, although painstakingly collected, it was obtained from just three GPCRs. This precludes using a neural network approach, as it would likely be biased towards GPCR stability. Instead, we extracted a linear scoring matrix, allowing us to observe trends in membrane protein stability and to devise a computationally-efficient tool that helped us stabilise three membrane proteins with different folds and modes of action. To apply a neural network approach that would work broadly with membrane proteins as has been done for GPCRs^56–58^ would require access to large systematic stabilisation datasets, with a record of both stabilising and destabilising variants of membrane proteins from diverse folds. We hope to increase the reporting, sharing and depositing of such data by releasing IMPROvER for use in the academic community http://improver.ddns.net/IMPROvER/.

IMPROvER lowers the experimental burden associated with discovering stabilising variants of membrane proteins. Thus, the upper limit of target amino acid length is much greater than previously used in scanning approaches: for example ClPPase, which we have successfully stabilised here, is dimeric and 700 residues long, and there is no intrinsic reason that IMPROvER cannot be used on membrane protein complexes. Furthermore, both the *deep-sequence* and *model-based* approaches of IMPROvER are not just restricted to alanine substitutions, broadening the scope of potential changes. Finally, we have shown that IMPROvER successfully stabilises helical multi-pass membrane proteins from diverse folds, because it uses bioinformatic information that can be extracted from any helical membrane protein. Thus, we have developed a general-purpose tool.

## Methods and materials

### Deep-sequence approach

The *deep-sequencing* module of IMPROvER builds on the EVmutations software^8^, a module of the larger EVcouplings package for evolutionary covariance analysis^7^. In the package, the UniProt90 database is iteratively searched by JackHMMER^59^, allowing the identification of distantly related sequences, an important aspect for making evolutionary comparisons. The EVcoupling module plmc is used to construct a matrix weighted scoring for each position: how alike each position in the query sequence is to the consensus. EVmutation is then used to make a prediction whether a position would be better if it were changed to one of the other amino acids, calculated by comparing the current amino acid at a position to the amino acid consensus from alignment. In this analysis, infrequently used, potentially detrimental amino acid side-chains are selected for substitution to more frequently used ones. The program is essentially looking for ‘odd-ones-out’. The selections may improve protein stability or may be beneficial in other ways (assisting expression, improving activity or speeding-up folding). No prior structural information is required. IMPROvER is currently configured to only take variants predicted to be stabilising with a normalised score above 70%.

### Model-based approach

In this module, the software package FoldX was used for in silico mutagenesis and free-energy calculations^9^. First, all input models were energy minimised by side-chain optimisation using the Repair module of FoldX. The models were then run through the AlaScan module of the FoldX, which estimates the $\Delta$G change (wild-type—variant) in unfolding energy for each position if it were changed to alanine. Positions were sorted based on the most negative average $\Delta \Delta$G change, i.e. the highest average stabilising effect. Positions that had a normalised score above 70% were mutated to every other amino acid using the PositionScan module of FoldX. To make calculations less computationally expensive, PositionScan was only implemented on the single best model (the model with lowest free energy of unfolding after minimisation). All output models from PositionScan were then energy minimised by side-chain optimisation using the Repair module of FoldX once again, removing bad clashes introduced by in silico mutagenesis. The energy of unfolding for these models were then compared to energy of unfolding for wild-type to assess $\Delta \Delta$G changes. Results for each position were sorted to select the best amino acid substitution for each of the selected sites.

### Data-driven approach

A GPCR dataset provided a single stability measurement for most of the positions in human adenosine $\hbox {A}_{{\mathrm{2A}}}$ receptor ($\hbox {A}_{{\mathrm{2A}}}\hbox {R}$), rat neurotensin receptor 1 (NR1) and turkey $\upbeta _{1}$ adrenergic receptor ($\upbeta _{1}\hbox {AR}$). Stability data for $\hbox {A}_{{\mathrm{2A}}}\hbox {R}$ were available in the apo, agonist and antagonist bound states, for NR1 in the agonist and apo states and for $\upbeta _{1}\hbox {AR}$ only in the apo state. We considered each of these sets of data to be independent as they are in different conformations, thus have differing tertiary structures. This provided just under 2,000 mutation data points, capturing both stabilising and destabilising changes. A stringent cut-off was applied when assigning stabilising or destabilising variants: above 140% of wild-type stability were assigned as stabilising and below 80% of wild-type stability as destabilising. Those in the destabilised category may also represent protein with reduced expression levels. Importantly, these datasets only inform on the most likely positions for stabilisation when changed to alanine (or leucine if the native residue was alanine) as this was the information provided by the GPCR datasets.

Each of the input GPCRs were run through the following bioinformatics tools: PsiPred for secondary structure prediction^14^, DisoPred for disorder prediction, MemSatSVM for membrane topology^60^, RYTHEM for lipid and helix contact prediction^61^, and JackHMMER^59^ for multiple sequence alignment and conservation extraction.

The data were weighted to allow comparison between the different bioinformatic factors. This was done by applying abundance weighted scoring: the frequency with which a factor was associated with stabilising or destabilising effects and internally normalised to the total number of observations for that factor. This reduced the skew towards common factors, allowing better comparisons between the stabilising properties of, for example, highly abundant (e.g. alanine) and rare residues (e.g. tryptophan) (Fig. S1). We were able to derive a simple scoring matrix in which factors least associated with destabilising changes and most associated with stabilising changes scored the highest. For example, glutamine was the 4th amino acid most associated with stabilising changes (score of 17/20), and was the amino acid least associated with destabilising changes (score of 20/20), providing an overall score of 37 (Table S1).

This scoring method forms the basis used by the *data-driven* module of IMPROvER to rank positions. A final score was given to each position in the protein, calculated as the product of multiplying scores of five bioinformatic factors together (Table S1): (1) amino acid identity, (2) topology, (3) conservation, (4) lipid contact prediction and 5) helix contact prediction. Each individual factor was trialled for predictive power before using them in combination, and using them in combination was the most powerful predictive method (Fig. 2A,B). Disorder prediction was not included at the time of testing in vitro targets, but has been included in the final version IMPROvER as overall prediction is slightly increased when included against the GPCR test set. The baseline for random selections of variants was established as 7.6 (± 1.0; SD n $=$ 10) stabilisers or 48.5 (± 5.9; SD n $=$ 10) destabilisers per 100 amino acids by ten random samplings of the input data.

### Primer design

The primers designed for this study, and the ones that IMPROvER automatically creates, are non-overlapping primers for inverse PCR site-directed mutagenesis^62^. They create a PCR product of the whole plasmid, where the ends are joined by blunt-end ligation. The primer melting temperature calculation used for selecting primer length is based on the “nearest-neighbour” method^63^, which takes experimentally derived values for the energy required to melt a given 2 bp stretch, and estimates a whole primer in terms of Gibbs free energy required Eq. (1).

1 $$\begin{aligned} primer \, melting \, temperature = \frac{(\Delta H_i^{\circ} + H^{\circ}) \times 1000}{ \Delta S_i^{\circ} + \Delta S^{\circ} + R \times \ln C_p } - 273.15 \end{aligned}$$

where $C_p$ is the primer concentration, $H^{\circ}$ is enthalpy (cal mol$^{-1}$), $\Delta S^{\circ}$ is entropy (cal K$^{-1}$ mol$^{-1}$) and *R* is the universal gas constant (1.987 cal K$^{-1}$ mol$^{-1}$)

Forward primers were designed to have at least 10 bps either side of the mismatch, but more on the 3′ side depending on primer melting temperature. The primer is iteratively minimised to an ideal melting temperature between 65 and 70 $^{\circ }$C by adding or removing bases from the 3′ end. The reverse primer is iteratively minimised to match the melting temperature of the forward primer. Primers are then subjected to further optimisation, refining the melting temperature as close to 62 $^{\circ }$C as possible so that a single annealing temperature can be used for all mutations during PCR amplification.

### Gene constructs and mutagenesis

Unless stated otherwise, enzymes and buffers for molecular cloning were obtained from New England Bioscience (NEB). For studies of ClPPase, the protein coding region for the putative $\hbox {K}^{+}$-stimulated pyrophosphate-energised dual $\hbox {Na}^{+}/\hbox {H}^{+}$ pump from *Clostridium leptum* (UniProt: A7VNH8) was made synthetically with the addition of an N-terminal 8xHis-tag and superfolder GFP followed by a TEV protease cleavage site (ENLYFQ). This construct was transferred to a pDDGFP yeast expression plasmid^64^ by homologous recombination, displacing an existing yGFP tag in the backbone and putting the gene under the control of the galactose promoter. For studies of hENT1, a pFastBac plasmid containing the hENT1 protein coding sequence from the gene *slc29a1* (UniProt: Q99808), which had been C-terminally tagged with eGFP-His6 and separated by a TEV protease cleavage site (ENLYFQ) was obtained from previous studies^65^. For studies of $\hbox {PTH}_{1}\hbox {R}$, the truncated human receptor (residues 180–593) was cloned into a pFastBac plasmid with a C-terminal GFP-His8 tag. To improve expression, the extracellular domain was replaced with apocytochrome b562RIL (BRIL) using an In-Fusion HD Cloning Plus kit following the manufacturer’s protocol (Takara-Bio).

For inverse PCR to amplify whole plasmids, $$5\,\upmu \hbox {L}$$ of 2 $$\times$$ Q5 polymerase pre-mix was mixed with $$1\,\hbox {ng }\,\upmu \hbox {L}^{-1}$$ template DNA, $$0.5\,\upmu \hbox {M}$$ of forward and $$0.5 \,\upmu \hbox {M}$$ of reverse primer, and made to a final volume of $$10 \,\upmu \hbox {L}$$ in nuclease free $$\hbox {H}_{2}\hbox {O}$$. The reaction was performed using a T100 thermocycler (Bio-Rad) with the following program: initial heat activation at 98 $$^{\circ }$$C for 5 min, cycling through 30 s of denaturation at 98 $$^{\circ }$$C, 30 s of annealing at 65 $$^{\circ }$$C and 3 min of extension at 72 $$^{\circ }$$C 30 times, followed by a final extension for 5 min at 72 $$^{\circ }$$C and holding at 8 $$^{\circ }$$C. PCR products were then subjected to blunt-end ligation in the presence of DpnI restriction enzyme by mixing a 2:1:1:1 ratio of DpnI, T4 DNA ligase, T4 polynucleotide kinase and 10 $$\times$$ T4 DNA ligase buffer, respectively, adding $$2\,\upmu \hbox {L}$$ of this mixture to $$10\,\upmu \hbox {L}$$ of PCR product and incubating for 1 h at 20 $$^{\circ }$$C. $$2\,\upmu \hbox {L}$$ of this reaction was then transformed into chemically competent OmniMax cells by heat-shock (20 min at 4 $$^{\circ }$$C, 45 s at 42 $$^{\circ }$$C and 2 min at 4 $$^{\circ }$$C) in 96-well PCR plate format for high-throughput. Following heat-shock, cells were recovered at 37 $$^{\circ }$$C for 1 h in LB medium before being spread on LB agar plates containing $$50\,\upmu \hbox {g}$$$$\hbox {mL}^{-1}$$ carbenicillin. After 16 h at 37 $$^{\circ }$$C, colonies were picked for overnight culture in 4 mL LB medium with $$50\,\upmu \hbox {g mL}^{-1}$$ ampicillin. Plasmids were then mini-prep isolated (Machery Nagel) and DNA sequencing (Eurofins) over the region of mutagenesis to confirm correctly incorporated variants. Some mutations required multiple rounds of reactions before successful site mutation was confirmed.

### Yeast culture and expression of ClPPase

The *S. cerevisiae* strain FGY217 (genotype: *MAT*$\upalpha$, *ura3-52, pep*$\Delta$4, *lys2*$\Delta$201)^66^ was incubated at 30 $^{\circ }$C for 72 h on YPD plates (2% (w/v) peptone, 1% (w/v) yeast extract, 2% (w/v) d-glucose, 2.5% (w/v) agar). Per transformation, 1 mL YPD media was inoculated with a colony grown on the YPD plate and incubated at 30 $^{\circ }$C overnight. Cells were then harvested by centrifugation at 3,000$\times$*g* for 5 min and washed in 1 mL sterile ultra pure $\hbox {H}_{2}\hbox {O}$ three times. During the final washing step, the cell suspension was split into 1 mL aliquots prior to discarding the supernatant. The cell pellet was resuspended in $360\,\upmu \hbox {L}$ sterile transformation mixture containing 10 mM lithium acetate, 33.3% (w/v) PEG 3350, $100\,\upmu \hbox {g}$ denatured fish sperm DNA and 100–500 ng plasmid. Heat-shock was carried out at 42 $^{\circ }$C for 40 min to trigger DNA uptake by the cells. Cells were centrifuged at 3,000$\times$*g* for 5 min and the pellet was resuspended in $200\,\upmu \hbox {L}$ of sterile $\hbox {H}_{2}\hbox {O}$. The resuspension was spread onto an SCD-Ura plate (0.67% (w/v) yeast nitrogen base, 2% (w/v) d-glucose, 2% (w/v) agar, $0.1 \hbox { mg mL}^{-1}$ carbenicillin, $105 \hbox { mg mL}^{-1}$l-threonine, $26 \hbox { mg mL}^{-1}$l-phenylalanine, $16 \hbox { mg mL}^{-1}$l-lysine, $10.5 \hbox { mg mL}^{-1}$ of l-arginine, $10.5 \hbox { mg mL}^{-1}$l-histidine, $10.5 \hbox { mg mL}^{-1}$l-leucine, $10.5\,\hbox {mg mL}^{-1}$l-methionine, $10.5 \hbox { mg mL}^{-1}$l-tryptophan, $10.5 \hbox { mg mL}^{-1}$l-tyrosine, $10.5 \hbox { mg mL}^{-1}$l-adenine) and incubated for 3 days at 30 $^{\circ }$C. A single colony was transferred to 5 mL of SCD-Ura media and incubated at 30 $^{\circ }$C for 16 h. This starter culture was used to inoculate 15 mL SCD-Ura media for single-temperature GFP-based stability assays or 100 mL SCD-Ura media for ten-temperature stability assay at an OD600 of 0.15. The expression cultures contained 0.1% d-glucose instead of 2% d-glucose and were incubated at 30 $^{\circ }$C for 5–6 h. ClPPase expression was induced at an OD600 of 0.6 by addition of 2% d-galactose and allowed to continue for 15 h at 30 $^{\circ }$C. Cells were harvested by centrifugation at 3,000$\times$*g* for 5 min.

### Stability testing of ClPPase

Cell pellets were resuspended in $750\,\upmu \hbox {L}$ lysis buffer (200 mM tris-HCl pH 7.5, 40% (w/v) glycerol, 10 mM EDTA, 2 mM dithiolthreitol (DTT), $2\,\upmu \hbox {g mL}^{-1}$ pepstatin A, 0.2 mM PMSF) and transferred into 2 mL tubes containing approximately 1.3 g of glass beads (0.5 mm diameter). The tubes were placed in a 24-tube adapter (Qiagen Catalogue number: 13000-V1-24) for a Vortex-Genie 2 (Scientific Industries) and vortexed at maximum speed for 30 min at 4 $^{\circ }$C to lyse the cells. Cell debris and glass beads were subsequently removed by centrifugation at 8,000$\times$*g*, 4 $^{\circ }$C for one min. The membranes were harvested by centrifugation at 36,000$\times$*g*, 4 $^{\circ }$C for 1 h and the pellet was solubilised in a total volume of $150\,\upmu \hbox {L}$ for a single point GFP-fluorescence-based stability assay, or $750\,\upmu \hbox {L}$ for a full ten-temperature GFP-fluorescence-based melting curve in 50 mM MES pH 6.5, 20% (w/v) glycerol, 37.5 mM KCl, 3.75 mM $\hbox {MgCl}_{2}$, 1 mM $\hbox {Na}_{4}\hbox {P}_{2}\hbox {O}_{7}$, 1.34% (w/v) dodecylmaltoside (DDM), 1 mM DTT, $0.15\,\upmu \hbox {g mL}^{-1}$ pepstatin A, and 0.25 mM PMSF. Solubilisation took place at 4 $^{\circ }$C for 1.5 h in 1.5 mL tubes continuously mixed by rolling. Residual insoluble material was removed by centrifugation at 37,000$\times$*g*, 4 $^{\circ }$C for 1 h. The fluorescence signal of the supernatant was measured using a QFX Fluorometer (DeNovix) to confirm the presence of GFP-tagged protein. All samples were diluted to the lowest fluorescence reading obtained within a set of samples to allow direct comparison between them. For screening experiments using the single-temperature GFP-based stability assay, the samples were split into two $60\,\upmu \hbox {L}$ aliquots. One remained on ice and one was heated to 50 $^{\circ }$C for 10 min in a Thermomixer (Eppendorf). For melting curve recordings of wild-type and ClPPase variants, detergent-solubilised sample was split into 11 aliquots of $60\,\upmu \hbox {L}$. One aliquot remained on ice while the others were independently incubated at a range of different temperatures (20, 30, 40, 45, 50, 55, 60, 70, 80 or 90 $^{\circ }$C). Afterwards, the samples were placed on ice for 30 min prior to centrifugation at 20,000$\times$*g*, 4 $^{\circ }$C for 30 min. $40\,\upmu \hbox {L}$ of the supernatant was subsequently transferred into fresh tubes and mixed with $10\,\upmu \hbox {L}$ of 5 $\times$ SDS-PAGE loading buffer (10% (w/v) SDS, 100 mM DTT, 50% (w/v) glycerol, 500 mM tris and 0.05% (w/v) bromophenol blue). $15\,\upmu \hbox {L}$ of each sample was loaded onto 4–20% Mini-PROTEAN TGX gels (Bio-Rad) and run at 150 V for 1 h. ClPPase bands were visualised by in-gel fluorescence detection using the G:BOX (G:BOX Chemi XX6 with Blue LEDs; Syngene). Data is representative of up to three biological repeats for variant, and up to eight biological repeats for wild-type ClPPase (Table S2).

### Phosphate release assay of ClPPase

The hydrolytic activity of wild-type and variant ClPPase in the membrane was assessed using a colorimetric phosphate release assay as previously described^67, 68^. In brief, the resuspended membranes were diluted with buffer containing 50 mM MES pH 6.5, 20% (w/v) glycerol, 50 mM KCl, 5 mM $$\hbox {MgCl}_{2}$$, 1.33 mM DTT, $$0.2\,\upmu \hbox {g mL}^{-1}$$ pepstatin A and 0.335 mM PMSF to a total membrane protein concentration between 2 and $$4\,\hbox {mg mL}^{-1}$$. Assay solution A (17 mM ascorbic acid in ice-cold 0.5 M HCl) and B (6 mM ammonium-heptamolybdate in ice-cold ultra pure $$\hbox {H}_{2}\hbox {O}$$) were prepared fresh and stored on ice until use. Either $$5\,\upmu \hbox {L}$$ of diluted membranes or $$\hbox {P}_{\mathrm{i}}$$ standard at 0.2, 0.5, 2, 4, 6, 8 or 10 mM was mixed with $$35\,\upmu \hbox {L}$$ reaction mixture (171 mM tris-HCl pH 8, 7 mM $$\hbox {MgCl}_{2}$$, 286 mM KCl, 57 mM NaCl, 2.85 mM NaF, 0.01 mM Gramicidin) and transferred to PCR-tubes. Samples and standards were prepared in triplicate. They were pre-incubated to 30 $$^{\circ }$$C for 5 min before the addition of $$10\,\upmu \hbox {L}$$ 2 mM tetrabasic sodium pyrophosphate. The reaction was allowed to proceed for 10 min at 30 $$^{\circ }$$C before samples were cooled on ice to quench. As a negative control, $$5\,\upmu \hbox {L}$$ of diluted membrane was added to the reaction mixture treated the same as samples and standards, but membranes were added after the reaction had been quenched. After 10 min on ice, $$60\,\upmu \hbox {L}$$ of a 1:1 mixture of assay solution A and B was applied to each tube and the samples were kept on ice for a further 10 min. $$90\,\upmu \hbox {L}$$ of sodium arsenite solution in 2% acetic acid (68 mM tribasic sodium citrate, 154 mM sodium arsenite) was added and the tubes were incubated at room temperature for 1 hour. The absorbance at 860 nm was measured using a Spark microplate reader (Tecan). Specific protein concentration of wild-type or variant ClPPase expressed in the membranes was estimated by measuring eGFP fluorescent signal against an eGFP calibration curve recorded using a QFX Fluorometer (DeNovix). Specific activity was calculated as the amount of phosphate released per min for a known amount of wild-type or variant ClPPase in the membrane $$(\upmu \hbox {mol P}_{\mathrm{i}}$$$$\hbox {min}^{-1}$$$$\hbox {mg}^{-1}$$).

### Insect cell culture and expression of hENT1 and $\hbox {hPTH}_{1}\hbox {R}$

In general, hENT1 and $\hbox {hPTH}_{1}\hbox {R}$ variants in pFastBac plasmid were transferred to Bacmids following manufacturer’s recommendations (Bac-to-Bac baculovirus expression system; Invitrogen). Briefly, DH10Bac *E. coli* (Invitrogen) were heat shock transformed with pFastBac-hENT1 or $\hbox {hPTH}_{1}\hbox {R}$ variant plasmids using a 96-well PCR plate format for high-throughput. Following recovery in LB medium for 16 h, cells were spread on LB agar plates with $50\,\upmu \hbox {g mL}^{-1}$ kanamycin, $7\,\upmu \hbox {g mL}^{-1}$ gentamicin, $10\,\upmu \hbox {g mL}^{-1}$ tetracycline, 100 mM IPTG and 0.2% Bluo Gal (Invitrogen). Following 72 h incubation at 37 $^{\circ }$C, suspected white colonies were re-streaked onto fresh LB agar plates with the same antibiotics and left for a further 24 h to confirm disruption of the $\upbeta$-galactosidase gene (*lacz*) by successful homologous recombination into the bacmid DNA. Confirmed white colonies were selected for overnight culture in 2 mL LB with $50\,\upmu \hbox {g mL}^{-1}$ kanamycin, $7\,\upmu \hbox {g mL}^{-1}$ gentamicin and $10\,\upmu \hbox {g mL}^{-1}$ tetracycline. Bacmid DNA was then isolated from pelleted cells (10 min, 3,000$\times$*g*, 20 $^{\circ }$C) by re-suspension in 50 mM tris-HCl pH 8.0, 10 mM EDTA and $100\,\upmu \hbox {g mL}^{-1}$ RNaseA, cell lysis in 200 mM NaOH and 1% SDS, neutralisation in 4.2 M guanidinium-HCl and 0.9 M potassium acetate pH 4.8, clarification (10 min, 16,000$\times$*g*, 20 $^{\circ }$C), precipitation and washing in ice cold 40% isopropanol, pelleting (10 min, 16,000$\times$*g*, 4 $^{\circ }$C), final washing in ice cold 70% ethanol, and re-suspension in ultra pure $\hbox {H}_{2}\hbox {O}$.

Following isolation, bacmids were transfected into *Sf9* insect cells using an adherent cell culture protocol scaled for 24-well plate format for high-throughput as follows: fresh mid-log phase *Sf9* cells were diluted to a density of approximately $0.55 \times 10^{6}$ cells $\hbox {mL}^{-1}$ in Insect-XPRESS Protein-free media (Lonza). These cells were distributed in 2 mL aliquots into each well of a poly d-lysine coated 24-well flat-bottom cell culture plate (Sarstedt), and left to adhere to the surface for 30 min. XtremeGENE HP Transfection Reagent (Roche) was diluted 1:10 in Insect-XPRESS Protein-free media (Lonza) to $100\,\upmu \hbox {L}$ and bacmid DNA added to $10 \hbox { ng}\, \upmu \hbox {L}^{-1}$. This transfection mixture was incubated at room temperature for 30 min before being added to the adherent cells. The plate was sealed and incubated for 3 days at 27 $^{\circ }$C. Medium containing virus was removed from the top of the adherent cells to form the initial stock of virus. 2 mL fresh, pre-warmed Insect-XPRESS Protein-free media (Lonza) was added to the cells and the plate incubated for a further 3 days. The cells were then imaged using an EVOS FL fluorescence confocal microscope for confirmation of GFP expression and plasma membrane localisation.

Virus amplification cultures were set up to both increase the volume of virus available for further infections and to increase the viral titre. Fresh mid-log phase *Sf9* cells were diluted to 1.0 $\times$ $10^{6}$ cells $\hbox {mL}^{-1}$ in Insect-XPRESS Protein-free media (Lonza) and distributed in 2.5 mL aliquots into each well of a sterile 24-well deep-well block with round bottoms. To each well, $25\,\upmu \hbox {L}$ of the initial virus from adherent cultures was added to begin infection and viral amplification. Cultures were incubated at 27 $^{\circ }$C for 3 days on an orbital shaker at 450 rpm. Amplified virus was then harvested by aspirating the media from the cell pellets following centrifugation of the cultures at 1,000$\times$*g*, 4 $^{\circ }$C for 10 min in swing-out plate adaptors.

Expression cultures were set-up using fresh mid-log phase *Sf9* cells, diluted to a density of approximately 2.0 $\times$ $10^{6}$ cells $\hbox {mL}^{-1}$ in 2.5 mL or 25 mL of 27 $^{\circ }$C pre-warmed Insect-XPRESS medium (Lonza) for hENT1 or $\hbox {hPTH}_{1}\hbox {R}$, respectively. For hENT1, cultures were set-up in wells of a sterile 24-well deep-well block with round bottoms, whereas $\hbox {hPTH}_{1}\hbox {R}$ cultures were set up in 125 mL sterile culture vessels. For hENT1, $50\,\upmu \hbox {L}$ of the amplified virus was added to each well to begin infection and expression of the hENT1 variants in a high-throughput mode. For $\hbox {hPTH}_{1}\hbox {R}$, $10 \,\upmu \hbox {L}$ of V1 virus was added to each culture to begin infection and expression of the $\hbox {hPTH}_{1}\hbox {R}$ variants. Cultures were incubated at 27 $^{\circ }$C for 3 days with shaking on an orbital shaker at 450 rpm. Cells were then harvested by centrifugation at 1,000$\times$*g*, 4 $^{\circ }$C for 10 min, frozen and stored at − 80 $^{\circ }$C until use. Expression cultures were performed in four biological repeats for hENT1 or three biological repeats for $\hbox {hPTH}_{1}\hbox {R}$.

### Stability testing of hENT1 and $\hbox {hPTH}_{1}\hbox {R}$

Each insect cell pellet was resuspended in $300\,\upmu \hbox {L}$ of resuspension buffer (50 mM tris-HCl, pH 7.5, 1 $\times$ Protease Inhibitor Cocktail) for hENT1, or 1 mL of 1 $\times$ phosphate-buffered saline (PBS) with protease inhibitor cocktail. Cell resuspensions were solubilised by the addition of 1% (w/v) DDM, carried out with inversions for 1 h at 4 $^{\circ }$C. Solubilised protein was then isolated by centrifugation (21,000$\times$*g*, 4 $^{\circ }$C for 1 h). For screening experiments using the single-temperature GFP-based stability assay, the supernatants were split into two aliquots of $50\,\upmu \hbox {L}$ between two 96-well PCR plates (one well per hENT1 variant). One PCR plate remained on ice and one was heated to 50 $^{\circ }$C or 39 $^{\circ }$C (for hENT1 or $\hbox {hPTH}_{1}\hbox {R}$, respectively) for 10 min in a T100 thermocycler (Bio-Rad). Each independent PCR plate had at least one wild-type repeat for comparison across different rounds of testing. For ten-temperature melting analysis of wild-type, pellets from larger cultures (scaled up tenfold) were obtained and protein solubilised equivalently to single temperature assays. One $50 \,\upmu \hbox {L}$ aliquot remained on ice while the others were successively incubated at a range of different temperatures (30, 35, 40, 45, 50, 55, 60, 65, 70, 75, 80 or 85 $^{\circ }$C for hENT1, or 4, 34, 36, 38, 40, 42, 45, 54, and 60 $^{\circ }$C for $\hbox {hPTH}_{1}\hbox {R}$). Following heating, samples were cooled to 4 $^{\circ }$C and kept on ice until centrifugation at 21,000$\times$*g*, 4 $^{\circ }$C for 35 min. $40\,\upmu \hbox {L}$ of each supernatant was then transferred to a fresh well of a PCR plate and $10\,\upmu \hbox {L}$ of 5 $\times$ SDS-PAGE loading buffer added to each. $15\,\upmu \hbox {L}$ of each sample was loaded onto 4–20% Mini-PROTEAN TGX gels (Bio-Rad) and run at 150 V for 1 h. In-gel hENT1 or $\hbox {hPTH}_{1}\hbox {R}$ linked GFP-fluorescence was visualised using a G:BOX (G:BOX Chemi XX6 with Blue LEDs; Syngene). Data is representative of three biological repeats for single-temperature and ten-temperature assays, of which two sets of the ten-temperature assays were used for an additional technical repeat (n $=$ 5 total) (Table S4).

### Inhibitor binding assay of hENT1

The remaining insect cell pellet of hENT1 variants was used for inhibitor binding assays. Each cell pellet was resuspended in $$500\,\upmu \hbox {L}$$ of resuspension buffer (50 mM tris-HCl, pH 7.5, 1 $$\times$$ cOmpleteTM protease inhibitor cocktail). The optical density of each cell resuspension was measured at 600 nm to inform normalisation. A working stock of [Benzyl-3*H*]-nitrobenzylthioinosine (PerkinElmer) ([3*H*]-NBMPR) was prepared by diluting stock [3*H*]-NBMPR in resuspension buffer to a final concentration of $$2\,\upmu \hbox {Ci}$$$$\hbox { mL}^{-1}$$ (64 nM). The cell resuspensions were divided into ten $$50\,\upmu \hbox {L}$$ aliquots for three technical repeats of three assay conditions.

The nine assay aliquots were incubated in three conditions: (1) no inhibitor (−/−), (2) 32 nM [3*H*]-NBMPR ($+$/−), (3) 32 nM [3*H*]-NBMPR and $20\,\upmu \hbox {M}$ dipyridamole ($+$/$+$). All conditions were incubated at room temperature for 1 h. Samples were applied to a GF/B filter (Whatman) pre-equilibrated in washing buffer (50 mM tris-HCl, pH 7.5) on a vacuum manifold (Promega). The liquid was pulled through the filters under vacuum, and washed three times with 2 mL of buffer (50 mM tris-HCl, pH 7.5). Filters were incubated overnight at room temperature in 10 mL of Ultima Gold scintillant (PerkinElmer). Radioactive disintegrations from bound [3*H*]-NBMPR in samples were quantified in counts per min using a TriCarb scintillation counter (PerkinElmer) using 10-min reads, which were performed twice. Deduction of background (−/−) and non-specific binding ($+$/$+$) allowed for the determination of hENT1 variant specific binding, and specific radioactive signal was normalised for each sample using total in-gel GFP-signal for each hENT1 variant, and then scaled relative to wild-type binding.

### Statistical analysis

GFP signal intensity of hENT1, $\hbox {hPTH}_{1}\hbox {R}$ and ClPPase bands after single and ten-temperature stability assays was quantified using ImageJ^69^ (Figs. S2, S7). The $T_m$ of wild-type and variant protein was obtained by plotting the fluorescence signal, normalised to the on-ice sample, against temperature challenges and data fitting with a four-parameter dose-response curve (variable slope) by non-linear least-squares fitting in the python package *scipy.stats*. The inflection point of the fitted melting curve represents the $T_m$ at which half of the protein is denatured. The standard error of the mean (SEM) of $T_m$ and $\Delta T_m$ determination was calculated where n > 1 (Tables S2, S4, S6). Error propagation was factored in using Eq. (2).

2 $$\begin{aligned} SEM_{\Delta T_m} = \sqrt{ (SEM_{\Delta T_{m \, WT}})^2 + (SEM_{\Delta T_{m \, variant}})^2 }. \end{aligned}$$

## Supplementary information

Supplementary Informations.

## Data Availability

Data generated in this study will be made available upon request to the authors. The IMPROvER pipeline is available for academic users by visiting http://improver.ddns.net/IMPROvER/. For commercial use of IMPROvER, users will be asked to obtain a licence from the University of Leeds.
