# Supplementary material for: IMPROvER: the Integral Membrane Protein Stability Selector
Source: Sci Rep. 2020 Sep 16;10:15165. doi: 10.1038/s41598-020-71744-x (PMC7495477; doi:10.1038/s41598-020-71744-x)
Supplement: Supplementary file 1 — Supplementary Informations. [file 41598_2020_71744_MOESM1_ESM.pdf]

IMPROvER: The Integral Membrane Protein Stability Selector
Supplementary Information

Steven P.D. Harborne[1,2,*,†], **Jannik Strauss**[1,†], **Jessica C. Boakes**[1,†], **Danielle L. Wright**[1], **James G. Henderson**[1], **Jacques Boivineau**[3], **Veli-Pekka Jaakola**[4], **and Adrian Goldman**[1,5,*]

[1]Astbury Centre for Structural and Molecular Biology, University of Leeds, UK
[2]Peak Proteins, BioHub, Alderley Park, Macclesfield, UK
[3]Novartis Institutes for Biomedical Research, Chemical Biology and Therapeutics, Virchow 16, Basel, Switzerland
[4]Confo Therapeutics, Technologiepark 94, 9052 Zwijnaarde, Belgium
[5]MIBS, Biological and Environmental Sciences, University of Helsinki, Finland

[*]Corresponding authors: steven.harborne@peakproteins.com and a.goldman@leeds.ac.uk
[†]these authors contributed equally

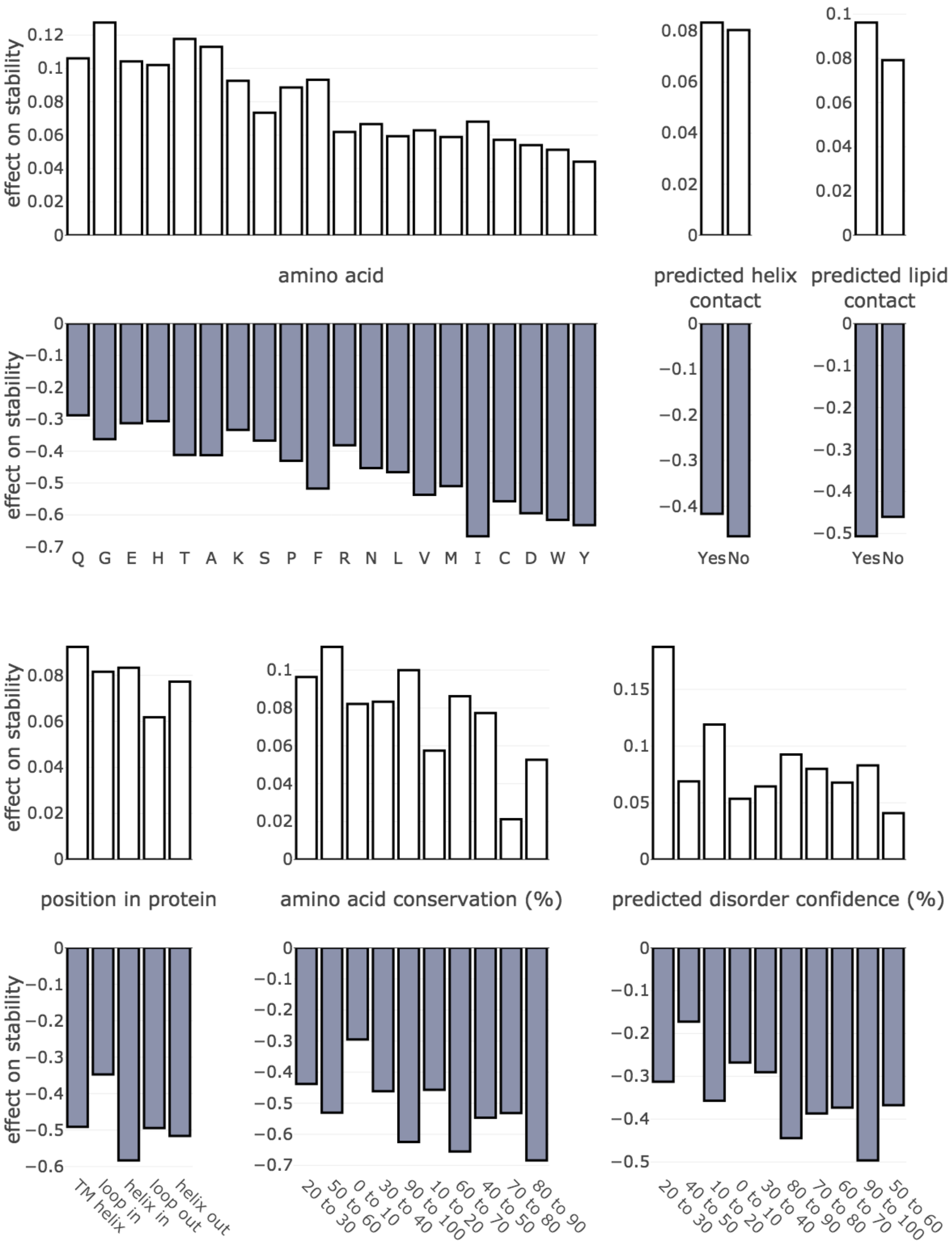

Figure S1. Analysis of stabilising versus destabilising trends in GPCR datasets. Analysis of stabilising versus destabilising trends in GPCR datasets when correlated with bioinformatic information. The frequency with which a factor was associated with stabilising or destabilising effects when changed to alanine (or leucine if natively an alanine) was calculated and normalised to the total number of observations of each factor. This provided a score for effect on stability for stabilisation (scale of 0 to 1) or destabilisation (scale of 1 to 0).

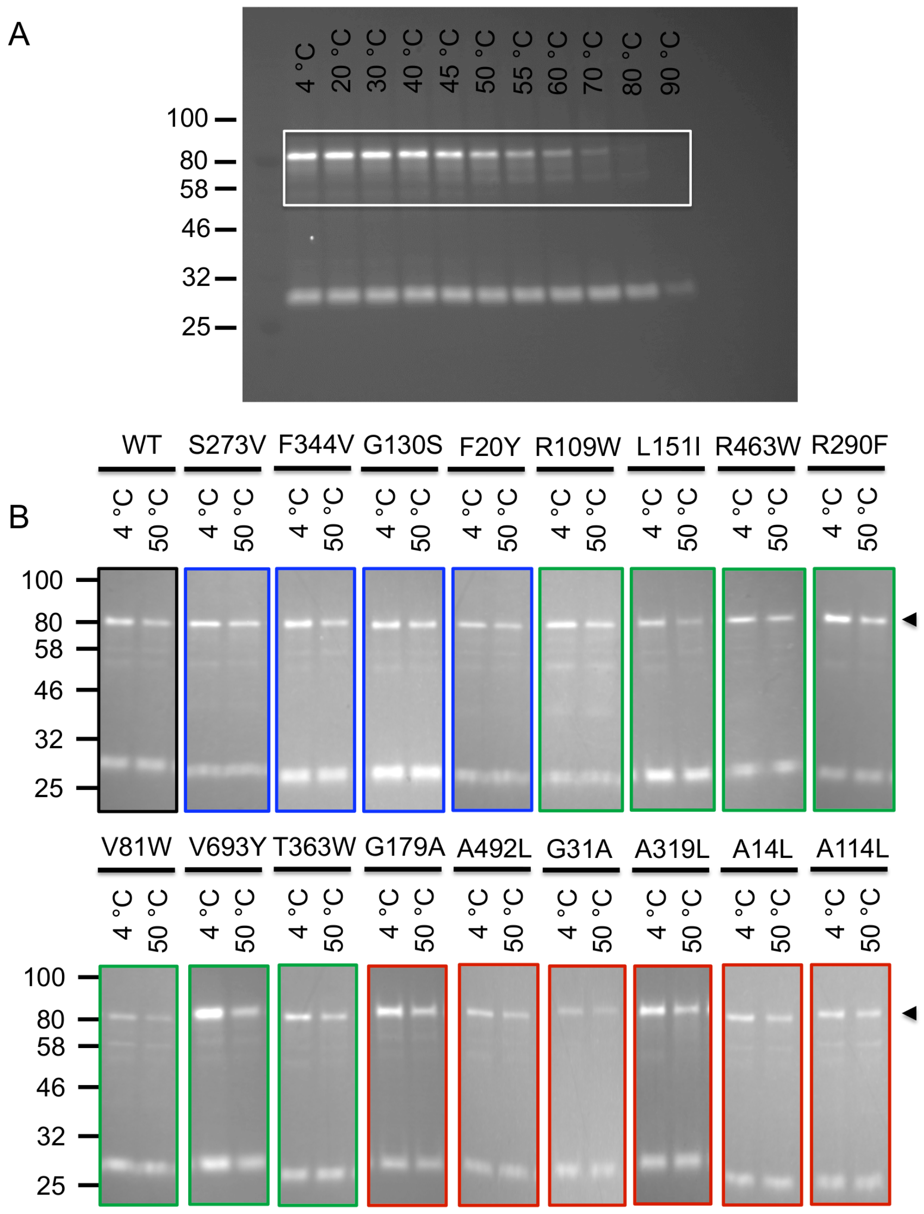

Figure S2. In-gel GFP-fluorescence analysis of ClPPase stability. A) Representative example of in-gel GFP-fluorescence from melting curve analysis of wild-type ClPPase. The white box denotes the cropped image used for the panel insert in Fig. 3D. B) Representative gels of single-temperature ClPPase stability assay. The band analysed is denoted by a black arrow. Outer edge colouring around each panel is according to the prediction module of IMPROvER from which the variant was selected (blue: *deep-sequence* based, green: *model-based*, red: *data-driven*).

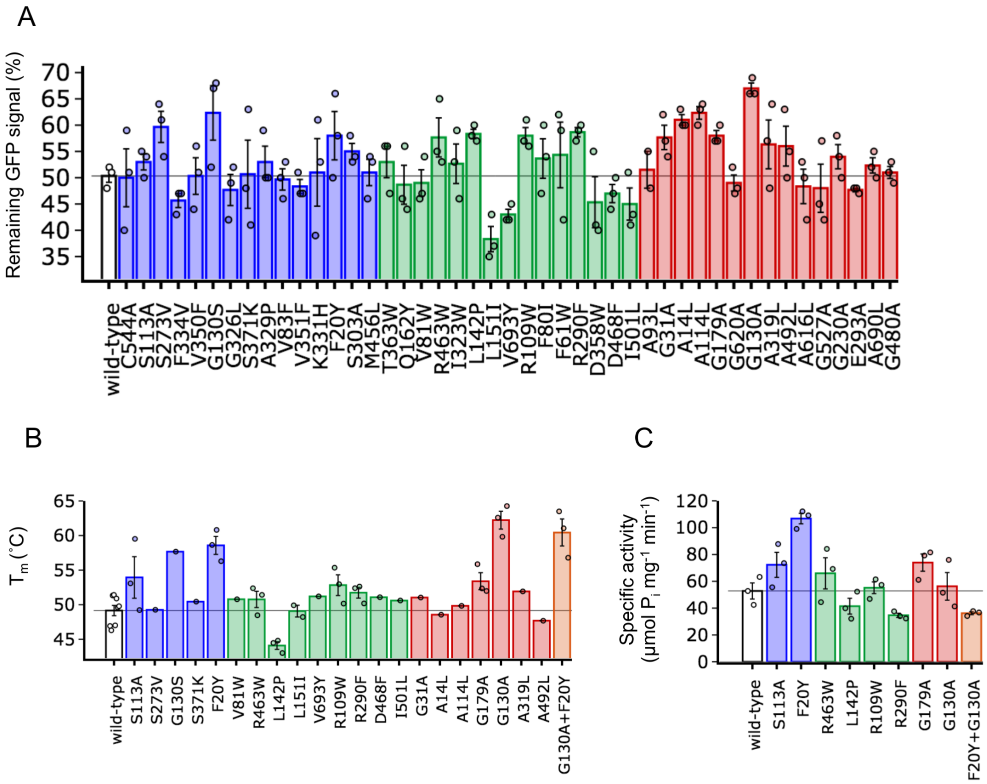

Figure S3. **Extended data for ClPPase variant stability and activity data.** A) Single-temperature stability assay results for ClPPase variants. Averages were derived from three biological repeats as indicated by open circles associated with each bar. B) Ten-temperature melting curve stability analysis results for select ClPPase variants. Values are derived from single experiments, or up to three repeats for variants (as indicated by open circles associated with each bar). Wild-type was tested eight times to provide a robust measurement for comparison. C) PP_i hydrolysis activity assay results for top stabilising and one destabilising (L142P) ClPPase variant. Values derived from three technical repeats. Colouring in all panels is according to the prediction module of IMPROvER from which the variant was selected (blue: *deep-sequence* based, green: *model-based*, red: *data-driven*). Orange has been used for the double F20Y/G130A variant. Error bars in all panels representative of the SEM.

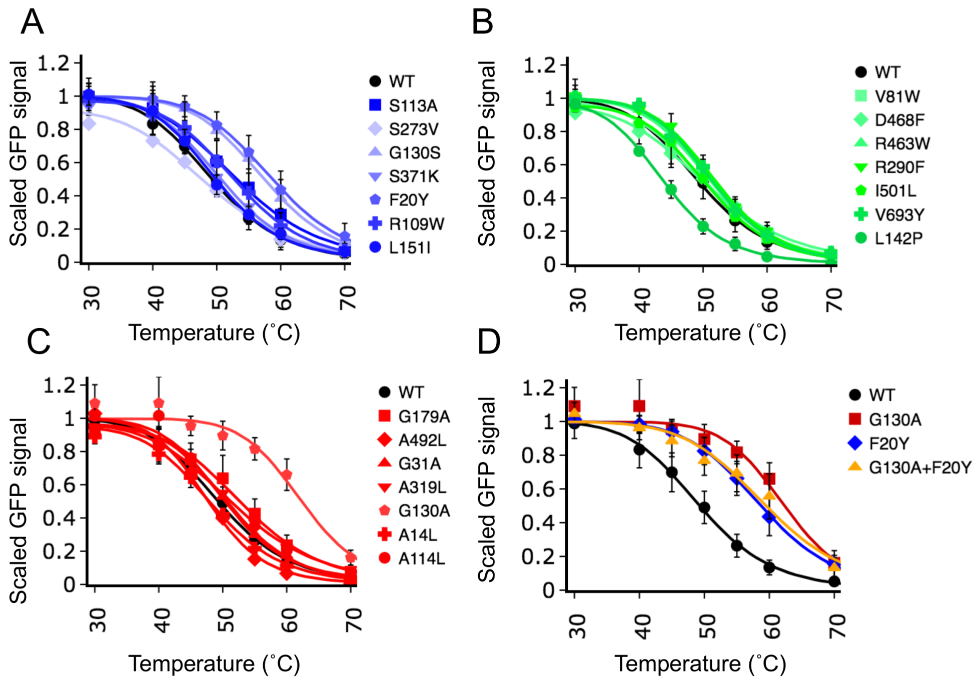

Figure S4. **Melting curves of ClPPase.** Protein that remained in solution after challenge in a ten-temperature melting curve was quantified using in-gel GFP fluorescence. Data for wild-type (black line) plotted with variants selected by the A) *deep-sequence* based (blue), B) *model-based* (green) or C) *data-driven* (red) modules of IMPROvER. D) The double variant of G130A+F20Y (orange) has been plotted relative to the single G130A (red) and F20Y (blue) varaints as well at wild-type (black). Three repetitions were done for the six best variants identified after recording the first round of melting curves for the selected variants. Error bars are representative of the standard deviation where repetitions were carried out. Data were fitted using a four-parameter dose-response curve (variable slope) by non-linear least-squares.

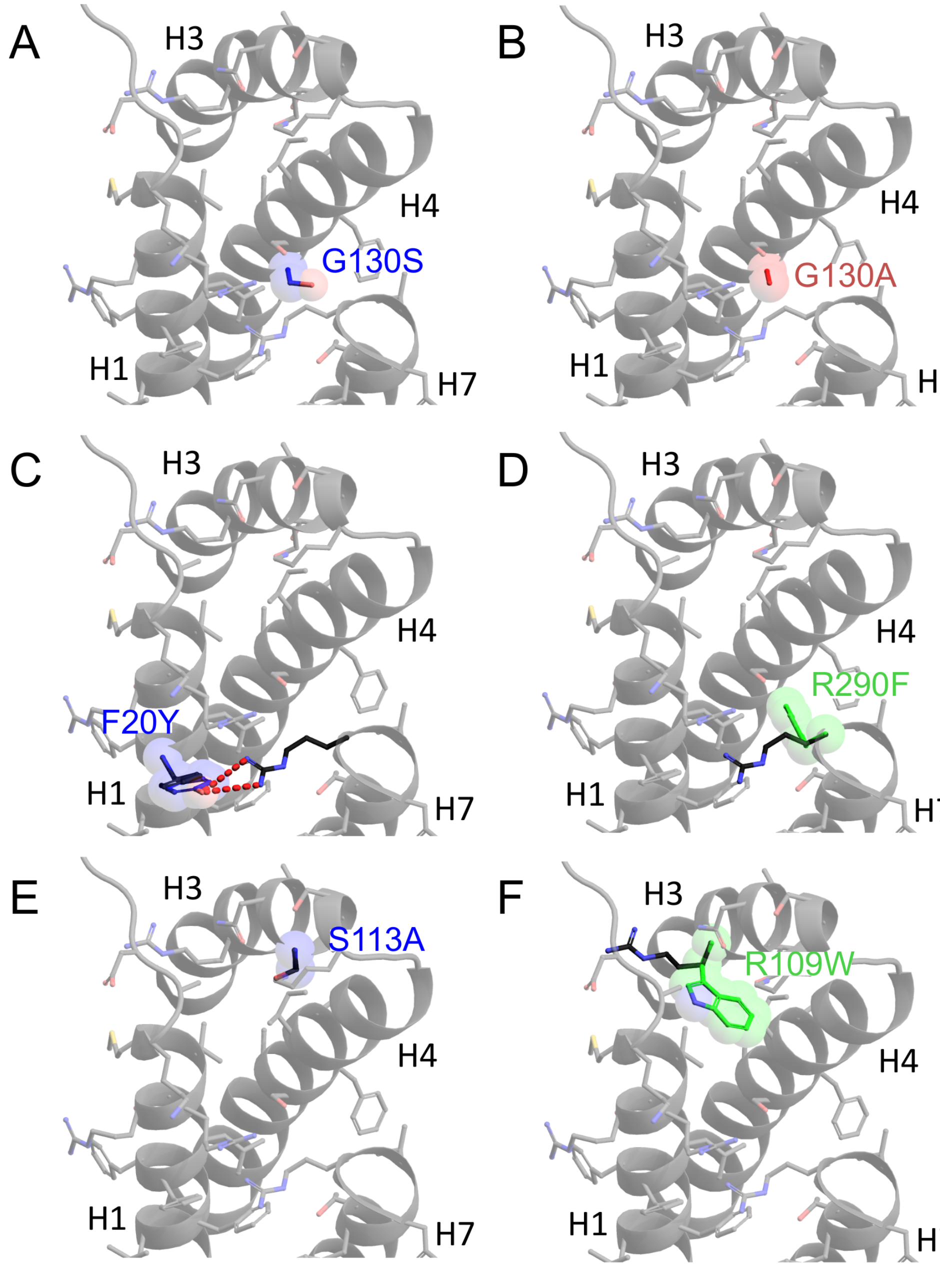

Figure S5. Structural rationalisation of stabilising ClPPase variants. A close-up view of helices (H) 1, 3, 4 and 7 in the best comparative homology model of ClPPase, representing a 'hot-spot' for stabilising mutations in ClPPase (all other helices removed for clarity). Six of the stabilising variants were located in this region: A) G130S, B) G130A, C) F20Y, D) R290F, E) S113A and F) R109W. Stabilising variants are shown in colour and space-filling spheres according to the prediction module of IMPROvER from which they were selected (blue: *deep-sequence* based, green: *model-based*, red: *data-driven*), with the native residue for the position or important interacting residues shown in black (all others shown in grey). Potential salt-bridge interactions are displayed as red dashed lines. Oxygen atoms are coloured red and nitrogen blue.

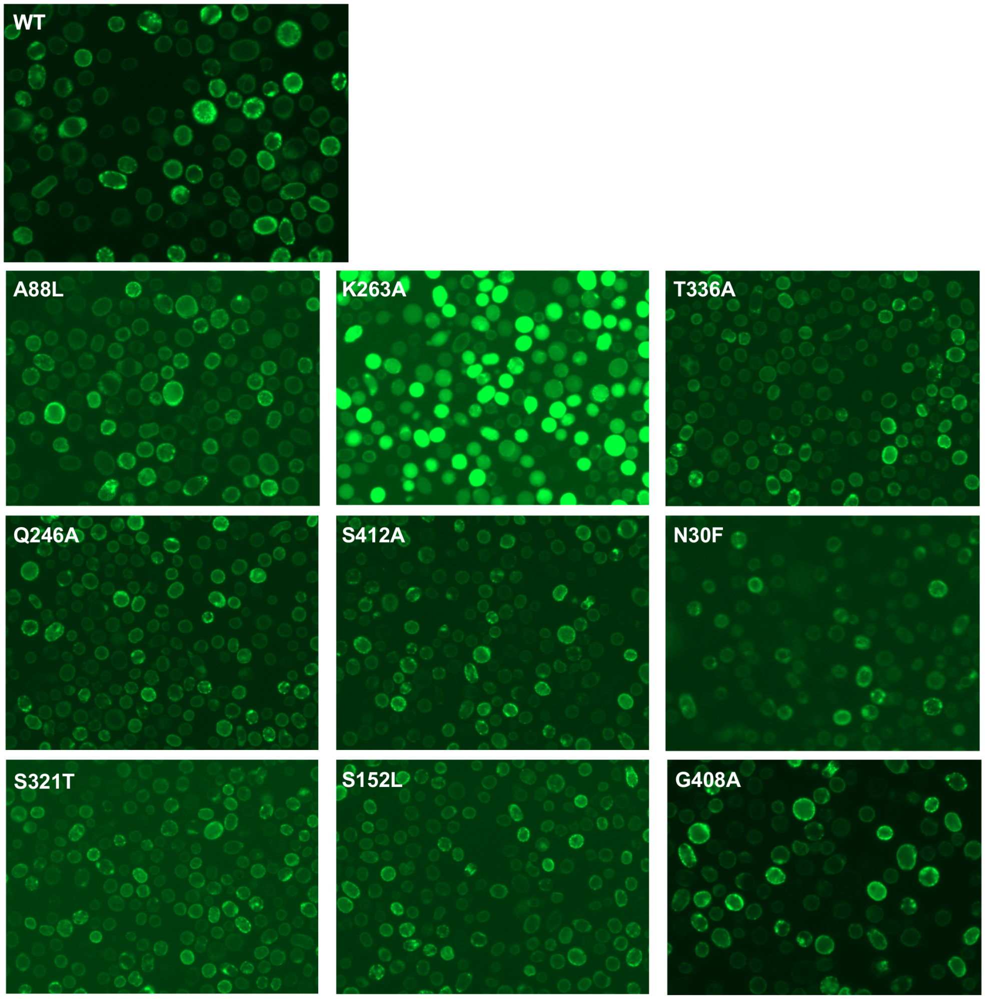

Figure S6. **In-cell GFP-fluorescence for hENT1 variants.** Representative example fluorescent confocal microscopy of GFP-signal from *Sf9* insect cells 3 days post baculovirus transfection. Examples of cells expressing wilt-type hENT1 and the hENT1 variants A88L, K263A, T336A, Q246A, S412A, N30F, S321T, S152L and G408A. The GFP signal in K263A appears greater than in the other cells, which could be due to increased protein expression levels but more likely due to an automatically selected longer exposure by the instrument. Images acquired with an EVOS FL fluorescence confocal microscope. Images produced at 20x magnification for all except WT and G408A, which are at 40x magnification.

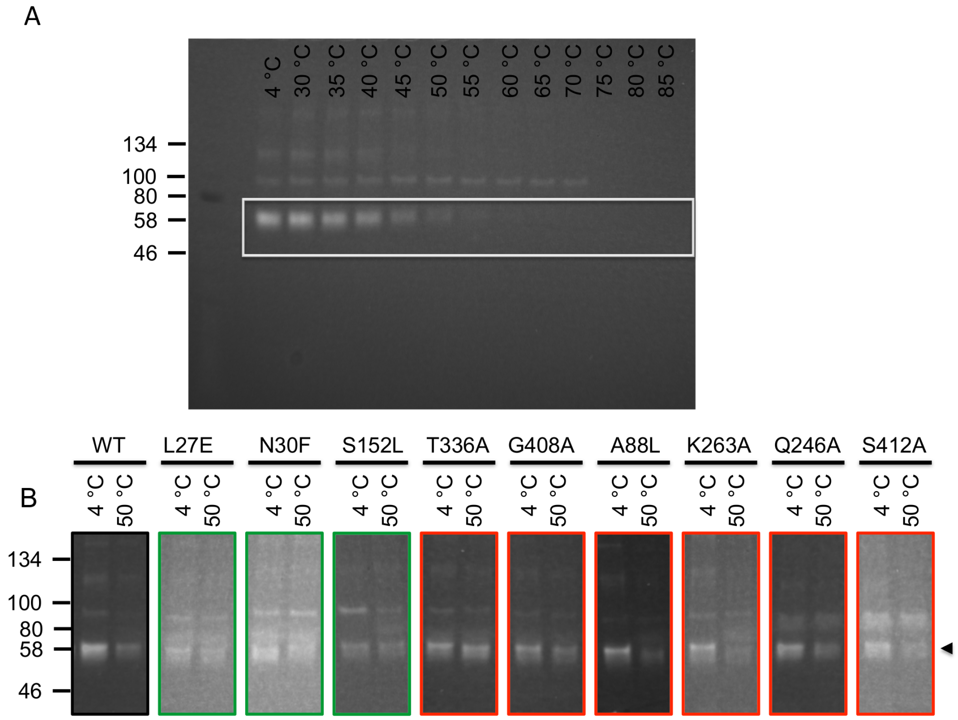

Figure S7. **In-gel GFP-fluorescence analysis of hENT1 stability.** A) Representative example of in-gel GFP-fluorescence from melting curve analysis of wild-type hENT1. The white box denotes the cropped image used for the panel insert in Fig. 4E. B) Representative gels of single-temperature hENT1 stability assay. The band analysed is denoted by a black arrow. Outer edge colouring around each panel is according to the prediction module of IMPROvER from which the variant was selected (blue: *deep-sequence* based, green: *model-based*, red: *data-driven*).

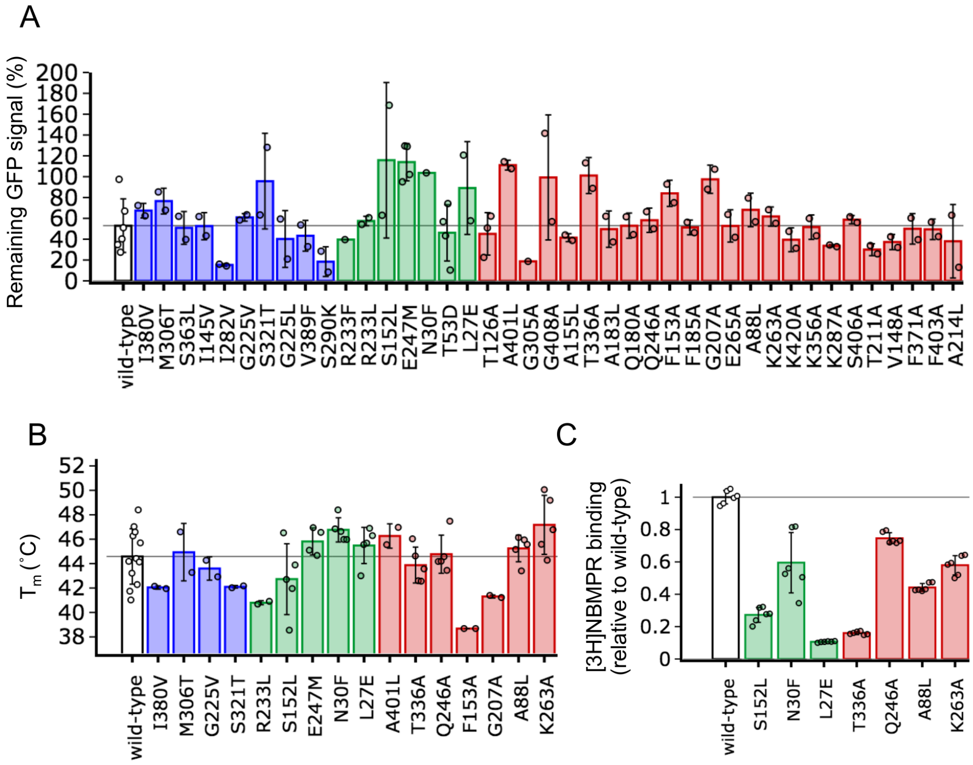

Figure S8. Extended data for hENT1 variant stability and ligand binding analysis. A) Single temperature stability assay results for hENT1 variants. Averages were derived from two or three biological repeats as indicated by open circles associated with each bar. B) Ten-temperature melting curve stability analysis results for selected hENT1 variants. Values are the average of five repeats for variants except for wild-type (as indicated by open circles associated with each bar). C) Binding of the radiolabelled hENT1 specific inhibitor NBMPR. Values are derived from the average of six readings from three biological repeats (two from each). Colouring in all panels is according to the prediction module of IMPROvER (blue: *deep-sequence* based, green: *model-based*, red: *data-driven*). Error bars in all panels representative of the SEM.

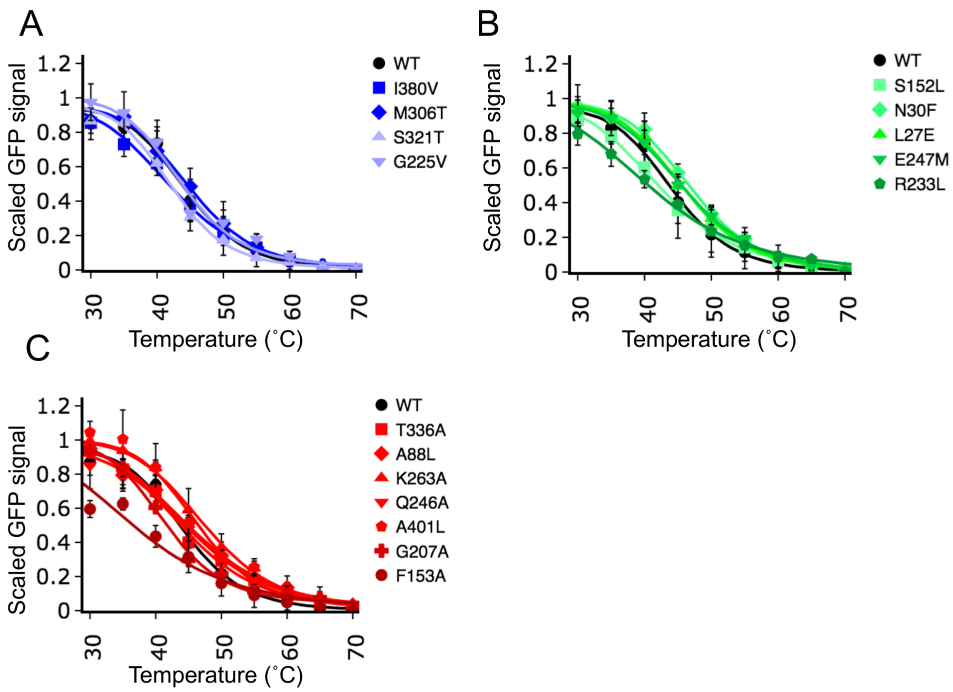

Figure S9. Melting curves of hENT1. Protein that remained in solution after challenge in a ten-temperature melting curve was quantified using in-gel GFP fluorescence. Data for wild-type (black line) plotted with variants selected by the A) *deep-sequence*-based (blue), B) *model-based* (green) or C) *data-driven* (red) modules of IMPROvER. Error bars are representative of the standard deviation from five repetitions. Data were fitted using a four-parameter dose-response curve (variable slope) by non-linear least-squares fitting.

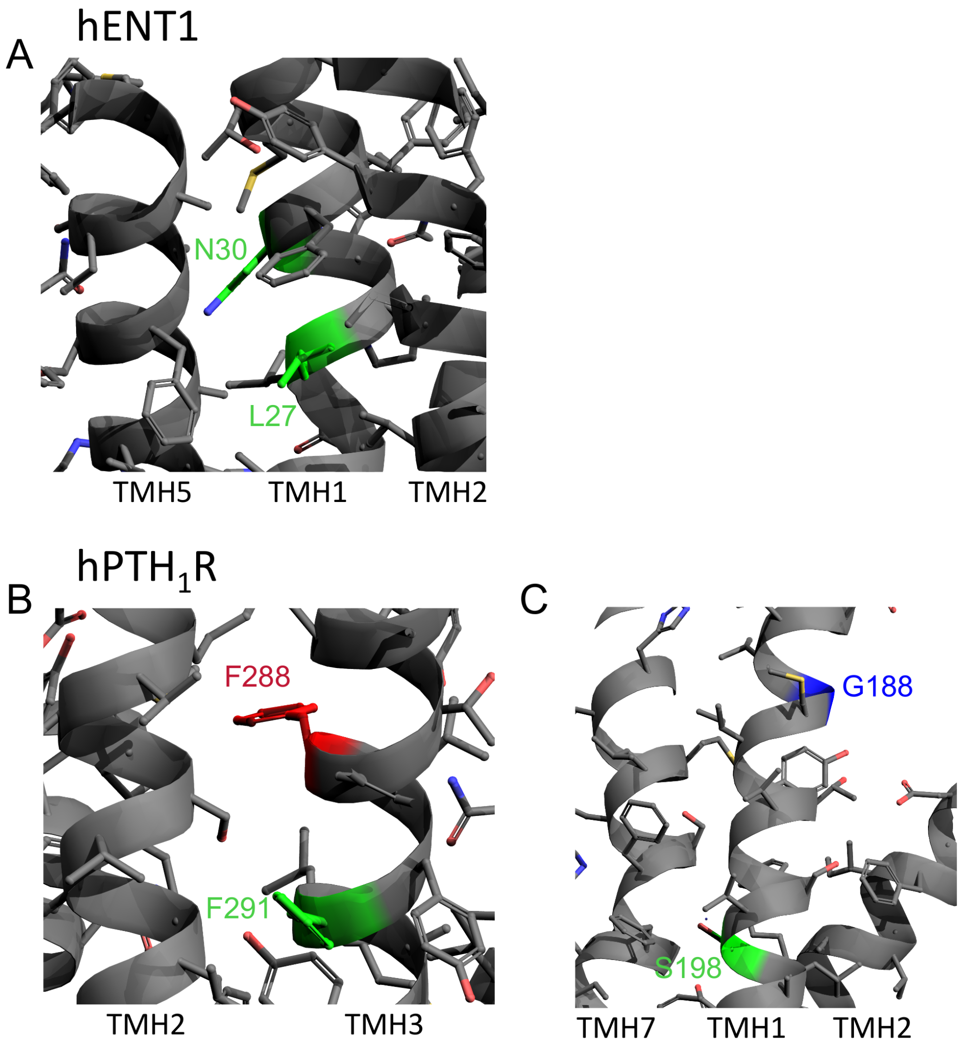

Figure S10. Structural rationalisation of stabilising hENT1 and hPTH$_1$R variants. A) Close-up view of transmembrane helices (TMH) 1, 2 and 5 to highlight the positions of the stabilising variants L27E and N30F in hENT1. B) Close-up view of TMH2 and TMH3 to highlight the positions of the stabilising variants F288A and F291T in hPTH$_1$R. C) Close-up view of TMH2 and TMH3 to highlight the positions of the stabilising variants F288A and F291T in hPTR$_1$. Stabilising variants are shown in colour according to the prediction module of IMPROvER from which they were selected (green: *model-based*, red: *data-driven*), with all other residues shown in grey. Oxygen atoms are coloured red, nitrogen blue and sulphur yellow.

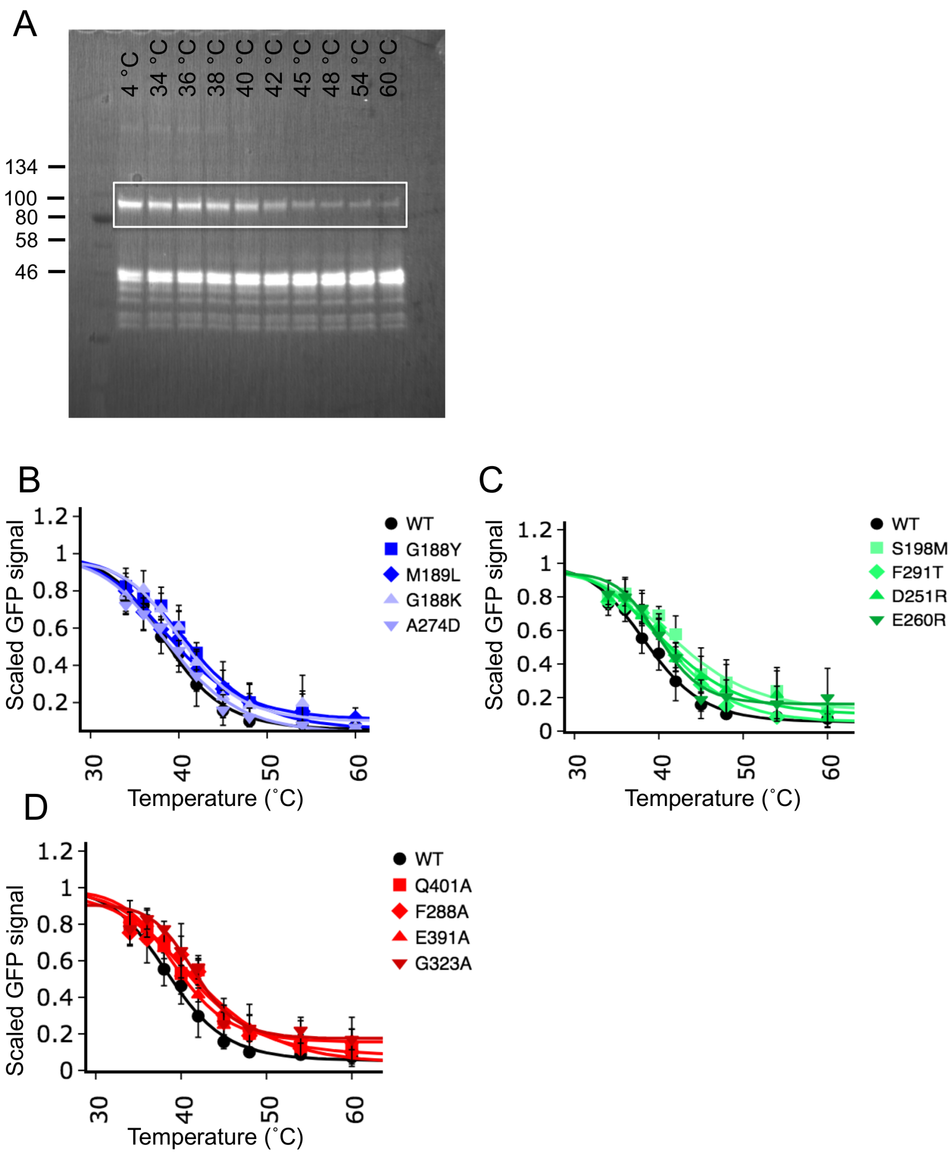

Figure S11. In-gel GFP-fluorescence analysis and melting curves of hPTH$_1$R. A) Representative example of in-gel GFP-fluorescence from melting curve analysis of wild-type hPTH$_1$R. The white box denotes the cropped image used for the panel insert in Fig. 5E. Protein that remained in solution after challenge in a ten-temperature melting curve was quantified using in-gel GFP fluorescence. Data for wild-type (black line) plotted with variants selected by the B) *deep-sequence*-based (blue), C) *model-based* (green) or D) *data-driven* (red) modules of IMPROvER. Error bars are representative of the standard deviation from three repetitions. Data were fitted using a four-parameter dose-response curve (variable slope) by non-linear least-squares fitting.

Table S1. Scoring matrix for data-driven approach of stabilising variant selection

Amino acid	Score
Q	37
G	36
E	34
H	34
T	32
A	30
K	30
S	26
P	23
F	21
R	21
N	19
L	15
V	14
M	13
I	11
C	9
D	7
W	5
Y	3

Lipid contact prediction	Score
Yes	3
No	2

Helix-helix contact prediction	Score
Yes	3
No	2

Topology	Score
TM helix	9
Cytosolic loop	8
Cytosolic helix	5
Extracellular loop	4
Extracellular helix	4

AA conservation (%)	Score
20 to 30	17
50 to 60	16
0 to 10	15
30 to 40	13
90 to 100	12
10 to 20	12
60 to 70	9
40 to 50	8
70 to 80	6
80 to 90	3

Disorder confidence score (%)	Score
20 to 30	17
40 to 50	15
10 to 20	15
30 to 40	11
0 to 10	11
80 to 90	10
70 to 80	9
90 to 100	8
60 to 70	8
50 to 60	6

Table S2. Table of residues selected by IMPROvER for ClPPase stabilisation

Module	IMPROvER Score	Variant	Sequence conservation (%)	Remaining GFP-signal (%)[a]	Error (±%)[b]	repeats (n)	T_m (°C)[c]	Error (±°C)[b]	repeats (n)	Comment
n/a	n/a	wild-type	n/a	50.3	1.2	3	49.3	0.9	8	n/a
deep-sequence	1.00	C544A	0	50.0	5.5	3	-	-	-	neutral
deep-sequence	1.00	S113A	2	53.0	1.5	3	53.6	2.8	3	stabilising
deep-sequence	1.00	S273V	1	59.7	3.0	3	48.8	-	1	neutral
deep-sequence	1.00	F334V	5	53.3	8.4	3	-	-	-	neutral
deep-sequence	0.99	V350F	12	50.3	3.5	3	-	-	-	neutral
deep-sequence	0.97	G130S	15	62.3	5.2	3	57.4	-	1	stabilising
deep-sequence	0.97	G326L	2	47.7	3.0	3	-	-	-	neutral
deep-sequence	0.97	S371K	9	50.7	6.5	3	50.6	-	1	neutral
deep-sequence	0.97	A329P	3	53.0	3.0	3	-	-	-	neutral
deep-sequence	0.96	V83F	25	49.7	2.0	3	-	-	-	neutral
deep-sequence	0.96	V351F	18	48.3	1.3	3	-	-	-	neutral
deep-sequence	0.95	K331H	4	51.0	6.4	3	-	-	-	neutral
deep-sequence	0.95	F20Y	11	58.0	4.6	3	58.4	1.2	3	stabilising
deep-sequence	0.95	S303A	8	55.0	1.5	3	-	-	-	neutral
deep-sequence	0.94	M456L	12	51.0	2.5	3	-	-	-	neutral
model-based	1.00	T363W	12	53.0	3.0	3	-	-	-	neutral
model-based	0.99	Q162Y	1	48.7	3.7	3	-	-	-	neutral
model-based	0.99	V81W	16	59.7	6.5	3	50.5	-	1	stabilising
model-based	0.99	R463W	27	57.7	6.0	3	51.1	1.3	3	neutral due to large error
model-based	0.99	I323W	11	52.7	3.8	3	-	-	-	neutral
model-based	0.98	L142P	75	41.5	16.5	2	44	0.5	3	destabilising
model-based	0.98	L151I	38	38.3	2.4	3	49.7	1.2	2	neutral
model-based	0.97	V693Y	15	43.0	1.0	3	51.4	-	1	stabilising
model-based	0.97	R109W	96	58.0	1.5	3	52.7	1.7	3	stabilising
model-based	0.96	F80I	11	53.7	3.8	3	-	-	-	neutral
model-based	0.95	F61W	8	54.3	6.2	3	-	-	-	neutral
model-based	0.95	R290F	34	58.7	0.9	3	52.0	0.6	3	stabilising
model-based	0.95	D358W	6	45.3	4.8	3	-	-	-	neutral
model-based	0.94	D468F	99	47.0	1.7	3	-	-	-	stabilising
model-based	0.94	I501L	18	45.0	3.1	3	50.6	-	1	neutral
data-driven	0.99	A93L	35	51.5	3.5	2	-	-	-	neutral
data-driven	0.98	G31A	82	57.7	2.3	3	51.0	-	1	stabilising
data-driven	0.96	A14L	54	61.0	1.0	3	48.4	-	1	neutral
data-driven	0.95	A114L	88	63.5	0.5	2	50.2	-	1	neutral
data-driven	0.91	G179A	60	58.0	1.0	3	52.9	1.5	3	stabilising
data-driven	0.90	G620A	96	49.0	1.5	3	-	-	-	neutral
data-driven	0.88	G130A	15	67.0	1.0	3	62.3	1.1	3	stabilising
data-driven	0.85	A319L	8	56.3	4.6	3	51.5	-	1	stabilising
data-driven	0.85	A492L	52	56.0	3.8	3	47.7	-	1	destabilising
data-driven	0.84	A616L	33	48.3	3.3	3	-	-	-	neutral
data-driven	0.79	G527A	51	49.5	9.0	3	-	-	-	neutral
data-driven	0.75	G230A	99	54.0	2.3	3	-	-	-	neutral
data-driven	0.71	E293A	25	47.7	0.3	3	-	-	-	neutral
data-driven	0.70	A690L	54	52.3	1.5	3	-	-	-	neutral
data-driven	0.68	G480A	94	51.0	1.2	3	-	-	-	neutral

[a] Calculated based on remaining in-gel GFP-signal after single-point temperature challenge thermostability assay.
[b] For n > 1: standard error of the mean (SEM) shown.
[c] Average T_m was calculated from individual T_m estimated for each individual repeat by fitting with a four-parameter dose-response curve (variable slope) by non-linear least-squares fitting in the python package *scipy.stats*

Table S3. Table of residues excluded from selection in ClPPase stabilisation

ClPPase Residue	PPase Study †	Mutation Studied	B-W ‡	Location	Role /Effect	Ref*
S173	TmPPase, AVP1, AVP1, ScPPase, VrPPase	M174, E229Q, E229D, K190R, E225	5.33	exit channel	facilitates ion release	1, 3, 3, 9, 13
L176	TmPPase, mPPase, ScPPase, ScPPase, VrPPase	S177, E193A, E193D, T228	5.36	exit channel	facilitates ion release	1, 7, 9, 9, 13
M180	TmPPase	L181	5.4	hydrophobic gate	prevent back-flow of ions?	1
R190	TmPPase, RbPPase, RbPPase, ScPPase, ScPPase, AVP1, AVP1, VrPPase, VrPPase	R191, R176A, R176K, R207A, R207K, R246A, R246K, R242, R242	5.42	coupling funnel	coupling of pumping and hydrolysis	1, 8, 8, 9, 9, 10, 10, 13, 13
K198	TmPPase, VrPPase, VrPPase	K199, K250A, K250	5.58	coupling funnel	coupling of pumping and hydrolysis	1, 11, 13
D231	TmPPase, VrPPase, VrPPase, RbPPase, RbPPase, ScPPase, VrPPase, RbPPase, RbPPase	D232, D283A, D283E, D217A, D217E, D248G, D283, D217A, D217H	-	-	loose coupling	1, 4, 4, 8, 8, 9, 13, 14, 14
D235	TmPPase, VrPPase, VrPPase, ScPPase, VrPPase	D236, D287A, D287E, D252G, D287	6.43	active site	coordination nucleophile	1, 4, 4, 9, 13
D242	TmPPase, ScPPase, VrPPase, VrPPase, VrPPase, VrPPase, VrPPase, VrPPase	D243, D259G, D294A, D294N, D294E, D294T, D294A, D294	6.5	ion-gate	ion-selectivity	1, 9, 11, 13, 13, 13, 13, 13
E245	TmPPase, RbPPase, RbPPase, ScPPase, ScPPase, ScPPase, VrPPase, ChlPPase, BvPPase, BvPPase, BvPPase	E246, E231Q, E231D, E262A, E262D, E262Q, G297A, E242D, E246A, E246Q, E246D	-	-	enhanced activity	1, 8, 8, 9, 9, 9, 10, 7, 16, 16, 16
S253	TmPPase, VrPPase, VrPPase, VrPPase	S254, A305S, A305, A305	6.61	exit channel	facilitates ion release	1, 10, 13, 13
A260	TmPPase, VrPPase, VrPPase	Y261, I312A, I312	6.68	exit channel	facilitates ion release	1, 10, 13
D484	TmPPase	D458	-	coupling funnel	coupling of pumping and hydrolysis	1
D445	TmPPase, VrPPase, VrPPase	D465, D507A, D507	11.57	active site	substrate/product binding	1, 11, 13
D472	TmPPase, VrPPase, VrPPase	N492, N534A, D534	12.43	active site	substrate/product binding	1, 11, 13
K479	TmPPase, RbPPase, RbPPase, RbPPase, RbPPase, BvPPase, BvPPase	K499, K469A, K469D, K469R, K469R, K489R, K489A	-	-	loose coupling/ low affinity	1, 8, 8, 8, 14, 16, 16

Continued on next page

Table S3 – *Continued from previous page*

ClPPase Residue	PPase Study †	Mutation Studied	B-W ‡	Location	Role /Effect	Ref*
A482	TmPPase, ScPPase, ScPPase, ScPPase, ScPPase, ScPPase, ScPPase, ScPPase, ScPPase, ScPPase, ScPPase, ScPPase, ScPPase, ScPPase, ScPPase, VrPPase	A502, A514S, A514L, A514I, A514M, A514P, A514Y, A514F, A514W, A514D, A514E, A514N, A514H, A514K, A514R, A544	12.53	coupling funnel	coupling of pumping and hydrolysis	1, 12, 12, 12, 12, 12, 12, 12, 12, 12, 12, 12, 12, 12, 12, 13
I483	TmPPase, VrPPase, VrPPase	I503, I545A, I545	12.54	coupling funnel	coupling of pumping and hydrolysis	1, 11, 13
A486	TmPPase, VrPPase	A506, A548	12.57	coupling funnel	coupling of pumping and hydrolysis	1, 13
A487	TmPPase, VrPPase	I507, A549	12.58	coupling funnel	coupling of pumping and hydrolysis	1, 13
L493	TmPPase, VrPPase, VrPPase, VrPPase	L513, L555A, L555D, L555	12.64	hydrophobic gate	prevent back-flow of ions?	1, 11, 11, 13
S496	TmPPase	S516	12.67	exit channel	facilitates ion release	1
S246	TmPPase, ScPPase, ScPPase, ScPPase, VrPPase, VrPPase, ChlPPase	S247, S263C, S263A, S263E, S298A, S298, S243A	6.54	nearby ion-gate	sodium binding impaired	1, 6, 9, 9, 10, 13, 7
K638	TmPPase, VrPPase	K663, K694	15.64	active site	substrate/product binding	1, 13
K639	TmPPase, VrPPase, VrPPase	K664, K695A, K695	15.65	active site	substrate/product binding	1, 11, 13
D661	TmPPase, VrPPase, VrPPase, VrPPase	D688, D723A, D723E, D723	16.31	active site	substrate/product binding	1, 4, 4, 13
D665	TmPPase, VrPPase, VrPPase, VrPPase	D692, D727A, D727E, D727	16.35	active site	substrate/product binding	1, 4, 4, 13
K668	TmPPase, VrPPase	K695, K730	16.38	active site	substrate/product binding	1, 13
D669	TmPPase, VrPPase, VrPPase, VrPPase, BvPPase, BvPPase	D696, D731A, D731E, D731, D704E, D704A	-	-	no activity	1, 4, 4, 13, 16, 16
N676	TmPPase, VrPPase, VrPPase, ChlPPase	D703, N738A, N738, N677D	16.46	nearby ion-gate	sodium binding impaired	1, 11, 13, 7
K680	TmPPase, VrPPase, VrPPase, VrPPase, VrPPase, ChlPPase	K707, K742A, K742R, K742M, K742, K681N	-	ion-gate	no activity	1, 13, 13, 13, 13, 7
M684	TmPPase, VrPPase, VrPPase, VrPPase	V711, V746A, V746D, V746	16.54	hydrophobic gate	prevent back-flow of ions?	1, 11, 11, 13
S691	TmPPase, VrPPase	S718, P753	16.61	exit channel	facilitates ion release	1, 13
F76	AVP1	E119Q	-	membrane cytosol interface TMH2	loose coupling	3
G249	AVP1, AVP1, VrPPase, VrPPase, VrPPase	E305Q, E305D, E301A, E301, E301	6.57	coupling funnel	coupling of pumping and hydrolysis	3, 3, 10, 13, 13

Continued on next page

Table S3 – *Continued from previous page*

ClPPase Residue	PPase Study †	Mutation Studied	B-W ‡	Location	Role /Effect	Ref*
E353	AVP1, AVP1, RbPPase, RbPPase, RbPPase	E427Q, E427D, E351D, E351A, E351Q	-	membrane cytosol interface TMH9	loose coupling/ low affinity	3, 3, 8, 8, 8
D438	AVP1, AVP1, RbPPase	D504N, D504E, D428N	-	membrane cytosol interface TMH11	no activity	3, 3, 8
T513	AVP1	D573N	-	loop 13-14	enhanced activity	3
N607	AVP1, VrP-Pase	E667Q, E663A	-	Transmembrane domain M15	loose coupling	3, 11
F685	AVP1	E751Q	-	C-terminal end	loose coupling	3
D201	VrPPase, VrPPase, ScPPase, VrPPase	D253A, D253E, D218G, D253	-	active site	substrate/product binding	4, 4, 9, 13
V207	VrPPase	V259A	-	-	loose coupling/ low affinity	4
K209	VrPPase, VrPPase, VrPPase	K261A, K261R, K261	-	active site	stabilising salt bridges?	4, 4, 13
E211	VrPPase, VrPPase, VrPPase, VrPPase, VrPPase	E263G, E263A, E263D, E268, E263	-	active site	stabilising salt bridges?	4, 4, 4, 13, 13
D227	VrPPase, VrPPase, ScPPase, VrPPase	D279A, D279E, D244G, D279	-	active site	substrate/product binding	4, 4, 9, 13
I252	VrPPase	C304R	-	-	loose coupling/ low affinity	4
A475	mPPase		12.46	active site	involved in K+ dependency	5
G478	mPPase		12.49	active site	involved in K+ dependency	5
V236	ScPPase	C253A	-	-	no activity	6
T369	ScPPase	S402C	-	-	no activity	6
E566	ScPPase	S609C	-	-	no activity	6
C578	ScPPase	C621A	-	-	no activity	6
S651	ScPPase	S694C	-	-	no activity	6
T87	mPPase		3.45	TMH3	dual pumping signature	7
F91	mPPase		3.49	TMH3	dual pumping signature	7
D143	mPPase		4.6	TMH4	dual pumping signature	7
R105	RbPPase	R101K	-	TMH3	enhanced activity	8
G192	RbPPase	G178A	-	TMH5	loose coupling/ low affinity	8
L387	RbPPase, AVP1, AVP1	E385Q, K461R, K461A	-	loop 9-10	pump-less	8, 10, 10
E585	RbPPase, RbPPase	E584A, E584D	-	TMH14	loose coupling/ low affinity	8, 8
S630	RbPPase	G637A	-	TMH15	loose coupling/ low affinity	8
T172	ScPPase	P189L	-	TMH5	pump-less	9
A174	ScPPase	V191A	-	-	loose coupling	9
T177	ScPPase	G194Q	-	-	loose coupling	9
F178	ScPPase	F195L	-	-	pump-less	9
G181	ScPPase	G198A	-	-	no activity	9
S183	ScPPase	A200T	-	-	loose coupling	9
M185	ScPPase	I202T	-	-	loose coupling	9
F188	ScPPase	F205S	-	-	loose coupling	9
G193	ScPPase	G210A	-	-	pump-less	9
G194	ScPPase	G211A	-	-	pump-less	9
D205	ScPPase, VrPPase	D222G, D257	-	active site	substrate/product binding	9, 13
L206	ScPPase	L223P	-	-	no activity	9
A212	ScPPase	Q229R	-	-	loose coupling	9
E216	ScPPase	E233G	-	-	loose coupling	9
I225	ScPPase	I242T	-	-	loose coupling	9
V229	ScPPase, ScPPase	V246I, V246A	-	-	loose coupling	9, 9
M239	ScPPase	M256T	-	-	loose coupling	9
Y244	ScPPase, VrPPase	F261L, F296A	-	TMH6	loose coupling	9, 10
G262	ScPPase, VrPPase	A278V, S314A	-	TMH6	low activity	9, 10
F265	ScPPase, VrPPase, VrPPase	D281G, L317A, L317A	-	Vacuolar lumen loop	low activity	9, 10, 11
P271	ScPPase	P287A	-	-	loose coupling	9

Continued on next page

Table S3 – *Continued from previous page*

ClPPase Residue	PPase Study †	Mutation Studied	B-W ‡	Location	Role /Effect	Ref*
I277	ScPPase	I293V	-	-	loose coupling	9
G278	ScPPase	G294R	-	-	no activity	9
T291	ScPPase	P307A	-	-	loose coupling	9
N299	ScPPase	S313E	-	-	loose coupling	9
L306	ScPPase	R320C	-	-	loose coupling	9
S311	ScPPase	S325R	-	-	no activity	9
L314	ScPPase	I328T	-	-	loose coupling	9
T308	ScPPase	F322L	-	-	loose coupling	9
A318	ScPPase	L332P	-	-	pump-less	9
L327	ScPPase	V351A	-	-	pump-less	9
G328	ScPPase	A357G	-	-	loose coupling	9
Y335	ScPPase, ScPPase	L368P, L368A	-	-	no activity	9, 9
G341	ScPPase	G374A	-	-	no activity	9
A344	ScPPase	L377P	-	-	pump-less	9
I348	ScPPase, ScPPase	I381P, I381A	-	-	loose coupling	9, 9
G349	ScPPase	Q382R	-	-	loose coupling	9
L243	VrPPase	L295A	-	TMH6	loose coupling	10
Y247	VrPPase	Y299A	-	TMH6	no expression	10
V248	VrPPase, BvPPase	A300S, C249S	-	TMH6	enhanced activity	10, 15
S250	VrPPase	S302A	-	TMH6	enhanced activity	10
I251	VrPPase	S303A	-	TMH6	low activity	10
T254	VrPPase	A306S	-	TMH6	no expression	10
A255	VrPPase	L307A	-	TMH6	no expression	10
A256	VrPPase	V308A	-	TMH6	loose coupling	10
L257	VrPPase	V309A	-	TMH6	low activity	10
A258	VrPPase	A310S	-	TMH6	loose coupling	10
A261	VrPPase	S313A	-	TMH6	low activity	10
L263	VrPPase	F315A	-	TMH6	low activity	10
G264	VrPPase, VrPPase	G316A, G316A	-	Vacuolar lumen loop	loose coupling	10, 11
K266	VrPPase	N318A	-	TMH6	no expression	10
T157	VrPPase	D218A	-	Vacuolar lumen loop	loose coupling	11
T197	VrPPase	T249A	-	Substrate binding site	no activity	11
D217	VrPPase, VrPPase	D269A, D269	-	active site	substrate/product binding	11, 13
N228	VrPPase	N280A	-	Substrate binding site	low activity	11
E698	VrPPase	E698A	-	Transmembrane domain M15	loose coupling	11
I687	VrPPase, VrPPase	L749A, L749D	-	Coupling funnel	no activity	11, 11
Y355	ScPPase, ScPPase, ScPPase, ScPPase, ScPPase, ScPPase, ScPPase	F388Y, F388G, F388P, F388D, F388E, F388K, F388R	-	-	no expression	12, 12, 12, 12, 12, 12, 12
D635	VrPPase	D691	-	active site	substrate/product binding	13
R553	VrPPase, TmPPase	R609, R578	13.62	TMH13	links motion of inner to outer ring	13, 13
S485	VrPPase	S547	-	coupling funnel	coordination of water	13
M189	VrPPase	L232	5.4	hydrophobic gate	prevent back-flow of ions?	13
E500	VrPPase	R562	12.71	exit channel	facilitates ion release	13
P374	RbPPase	H372A	-	TMH10	loose coupling/ low affinity	14
E645	RbPPase	H652A	-	-	loose coupling	14
H654	RbPPase	H661A	-	-	loose coupling/ low affinity	14
S695	RbPPase	H702V	-	-	loose coupling/ low affinity	14

† PPase abbreviations: AVP1; *Arabidopsis thaliana* (isoform 1) , BvPPase; *Bacteroides vulgatus*, ChlPPase; *Chlorobium limicola*, PaPPase; *Pyrobaculum aerophilum*, RbPPase; *Rhodospirillum rubrum*, ScPPase; *Streptomyces coelicolor*, TmPPase; *Thermotoga maritima*, VrPPase; *Vigna radiata*
‡ B-W; Ballesteros–Weinstein numbering scheme
* References: 1; Kellosalo et. al. 2012, 2; Harborne et. al. 2018, 3; Zhen et. al. 1997, 4; Nakanishi et. al. 2001, 5; Belogurov Lahti 2002, 6; Mimura et. al. 2004 , 7; Luoto et. al. 2013, 8; Schultz Baltscheffsky 2003, 9; Hirono, Nakanishi Maeshima 2007, 10; Pan et. al. 2011, 11; Asaoka, Segami Maeshima 2014, 12; Hirono Maeshima 2009, 13; Lin et. al. 2012, 14; Schultz Baltscheffsky 2004, 15; undergrad thesis Dovile Dormantaite 2016, 16; undergrad thesis Hannah Shephard 2016

Table S4. Table of residues selected by IMPROvER for hENT1 stabilisation

Module	IMPROvER Score	Variant	Sequence conservation (%)	Remaining GFP-signal (%)[a]	Error (±%)[b]	repeats (n)	T_m (°C)[c]	Error (±°C)[b]	repeats (n)	Comment
n/a	n/a	wild-type	n/a	53.0	10.5	6	44.6	0.6	12	n/a
deep-sequence	1.00	I380V	16	67.4	5.0	2	42.0	0.1	2	destabilisng
deep-sequence	0.96	M306T	2	76.6	8.8	2	44.9	0.1	2	neutral
deep-sequence	0.95	S363L	4	50.8	11.2	2	-	-	-	neutral
deep-sequence	0.92	I145V	18	52.5	9.2	2	-	-	-	neutral
deep-sequence	0.91	I282V	11	15.2	0.8	2	-	-	-	destabilisng
deep-sequence	0.91	G225V	2	60.9	2.5	2	43.6	0.7	2	neutral
deep-sequence	0.87	S321T	14	95.7	32.4	2	42.1	0.1	2	destabilisng
deep-sequence	0.87	G225L	2	40.1	19.2	2	-	-	-	neutral
deep-sequence	0.86	V389F	20	43.2	10.4	2	-	-	-	neutral
deep-sequence	0.86	S290K	5	18.4	14.2	2	-	-	-	destabilisng
model-based	0.99	R233F	38	39.6	-	1	-	-	-	destabilisng
model-based	0.99	R233L	38	57.4	3.2	2	40.8	0.1	2	destabilisng
model-based	0.99	S152L	20	115.7	52.8	2	42.7	1.3	5	neutral
model-based	0.98	E247M	19	113.9	9.0	2	45.8	0.6	2	stabilising
model-based	0.98	N30F	83	103.7	-	1	46.8	0.4	5	stabilising
model-based	0.97	T53D	2	50.0	6.8	2	-	-	-	neutral
model-based	0.96	L27E	56	89.2	31.5	2	45.5	0.7	5	stabilising
data-driven	1.00	T126A	43	45.1	20.4	2	-	-	-	neutral
data-driven	0.99	A401L	12	111.0	3.4	2	46.3	0.7	2	stabilising
data-driven	0.99	G305A	10	18.7	-	1	-	-	-	destabilisng
data-driven	0.98	G408A	94	99.2	60.0	1	-	-	-	stabilising
data-driven	0.98	A155L	38	41.4	2.6	2	-	-	-	destabilisng
data-driven	0.98	T336A	14	101.0	12.2	2	43.9	0.7	5	neutral
data-driven	0.98	A183L	49	49.6	12.3	2	-	-	-	neutral
data-driven	0.96	Q180A	56	53.0	8.6	2	-	-	-	neutral
data-driven	0.93	Q246A	4	58.2	8.2	2	44.8	0.7	5	neutral
data-driven	0.90	F153A	20	83.9	8.9	2	37.1	1.6	2	destabilisng
data-driven	0.90	F185A	6	51.3	5.0	2	-	-	-	neutral
data-driven	0.90	G207A	21	97.5	9.6	2	41.3	0.1	2	neutral
data-driven	0.87	E265A	5	52.8	11.0	2	-	-	-	neutral
data-driven	0.86	A88L	18	68.1	11.4	2	45.2	0.5	5	neutral
data-driven	0.81	K263A	2	61.8	6.5	2	47.2	1.1	5	stabilising
data-driven	0.80	K420A	33	39.4	8.1	2	-	-	-	destabilisng
data-driven	0.80	K356A	18	51.6	8.3	2	-	-	-	neutral
data-driven	0.80	K287A	45	33.6	0.8	2	-	-	-	destabilisng
data-driven	0.78	S406A	53	58.8	2.8	2	-	-	-	neutral
data-driven	0.70	T211A	46	29.9	4.0	2	-	-	-	destabilisng
data-driven	0.69	V148A	29	37.2	5.2	2	-	-	-	destabilisng
data-driven	0.67	F371A	71	49.9	10.4	2	-	-	-	neutral
data-driven	0.67	F403A	45	49.5	6.9	2	-	-	-	neutral
data-driven	0.63	A214L	12	38.0	25.0	2	-	-	-	destabilising

[a] Calculated based on remaining in-gel GFP-signal after single-point temperature challenge thermostability assay.
[b] For n > 1: standard error of the mean (SEM) shown.
[c] Average T_m was calculated from individual T_m estimated for each individual repeat by fitting with a four-parameter dose-response curve (variable slope) by non-linear least-squares fitting in the python package *scipy.stats*

Table S5. Table of residues excluded from selection in hENT1 stabilisation

ENT Studied	Mutation Studied	Role /Effect	Ref*
hENT1	W29G, W29C, W29A, W29Y, W29V, W29T	Altered permeant selectivity and reduced inhibitor sensitivity	7
hENT1	M33I, M33A	Altered permeant selectivity and reduced inhibitor sensitivity	8, 12
hENT2	I33M, I33C	Altered permeant selectivity and increased inhibitor sensitivity	
	I33A, I33S	Altered permeant selectivity and reduced inhibitor sensitivity	
hENT1	N48Q	Reduced inhibitor sensitivity	13
hENT1	M89C, M89T, M89V	Increased substrate transport and inhibitor sensitivity	10
	M89L, M89Q	Reduced inhibitor sensitivity	
hENT1	L92Q, L92P	Selective reduction in substrate transport and inhibitor sensitivity	6
hENT1	G154S	Reduced inhibitor sensitivity	11
LdNT1.1	S158C/ L465C	Reduced substrate transport	14
hENT1	S160C, S160N	Increased substrate transport and inhibitor sensitivity	10
LdNT1.1	T160A	Reduced substrate transport	14
LdNT1.1	Y161A	Reduced substrate transport	14
LdNT1.1	G162A, G162C/ S173C	Reduced substrate transport	14
LdNT1.1	M163A	Increased substrate transport	14
LdNT1.1	F164A	Increased substrate transport	14
LdNT1.1	F167A	Reduced substrate transport	14
LdNT1.1	G162C/ S173C	Reduced substrate transport	14
LdNT1.1	T174A	Reduced substrate transport	14
LdNT1.1	M175A	Reduced substrate transport	14
LdNT1.1	M176A, M176C/ M442C	Reduced substrate transport	14
hENT1	G179L, G179V, G179C	Reduced substrate transport and inhibitor sensitivity	1, 3
hENT1	G184L, G184V, G184C	Reduced substrate transport and inhibitor sensitivity	3
hENT1	F209A	Reduced substrate transport	1
hENT1	P308A	Reduced substrate transport	1
hENT1	F334C, F334Y, F334I, F334V, F334S	Altered permeant selectivity and inhibitor sensitivity	5
hENT1	N338S, N338C, N338M, N338D, N338D, N338Q, N338A	Altered permeant selectivity and inhibitor sensitivity	5
LdNT1.1	M176C/ M442C	Reduced substrate transport	14
LdNT1.1	L444A	Reduced substrate transport	14
LdNT1.1	V445A	Reduced substrate transport	14
LdNT1.1	L446A	Increased substrate transport	14
LdNT1.1	G447C/ G467C	Reduced substrate transport	14
LdNT1.1	S158C/ L465C	Reduced substrate transport	14
LdNT1.1	M466A	Reduced substrate transport	14
LdNT1.1	G467A, G447C/ 467C	Reduced substrate transport	14
LdNT1.1	I468A	Reduced substrate transport	14
LdNT1.1	S469A	Reduced substrate transport	14
LdNT1.1	I470A	Reduced substrate transport	14
LdNT1.1	L471A	Reduced substrate transport	14
hENT1	L442T, L442I	Altered permeant selectivity and inhibitor sensitivity	1, 7, 8

† ENT species abbreviations: Leishmania donovani ENT (LdNT1.1); human ENT (hENT).
* References: 1; Huang, W et al. 2017, 2; Osato, D.et al. 2003, 3; SenGupta, D. et al 2002, 4; Sundaram, M. et al 2001, 5; Visser F. et al 2007, 6; Endres, C. et al. 2004, 7; Paproski, R. et al 2008, 8; Visser F. et al 2005, 9; Aseervatham, V. et al 2015, 10; Endres, C. et al 2004, 11; Yao, S. et al. 2001, 12; Visser, F. et al. 2005, 13; Vickers, M. et al 1991, 14; Valdes, R. et al 2014

Table S6. Table of residues selected by IMPROvER for $hPTH_1R$ stabilisation

Module	IMPROvER Score	Variant	Sequence conservation (%)	Remaining GFP-signal (%)[a]	Error (±%)[b]	repeats (n)	T_m (°C)[c]	Error (±°C)[b]	repeats (n)	Comment
n/a	n/a	wild-type	n/a	54.0	2.2	3	38.7	0.8	3	n/a
deep-sequence	1.00	T427M	2	54.0	1.2	3	-		-	neutral
deep-sequence	1.00	A275K	4	47.5	12.2	3	-		-	neutral
deep-sequence	0.99	G188Y	1	64.6	1.0	3	41.2	0.7	3	stabilising
deep-sequence	0.99	M189L	3	62.3	1.4	3	39.1	0.9	3	neutral
deep-sequence	0.99	L228V	13	41.7	1.9	3	-		-	destabilise
deep-sequence	0.98	S356A	7	55.4	5.7	3	-		-	neutral
deep-sequence	0.98	G188K	1	64.0	2.1	3	40.3	1.2	3	stabilising
deep-sequence	0.98	A274D	1	64.6	6.3	3	39.1	0.1	3	neutral
deep-sequence	0.97	T427L	2	51.3	5.4	3	-		-	neutral
model-based	1.00	S198M	89	67.0	0.8	3	42.1	0.8	3	stabilising
model-based	0.99	F291T	38	65.8	2.2	3	40.9	0.9	3	stabilising
model-based	0.98	D251R	13	67.2	1.5	3	41.7	1.5	3	stabilising
model-based	0.98	E259P	3	51.4	4.0	3	-		-	neutral
model-based	0.97	E260R	1	62.1	1.6	3	39.7	1.5	3	neutral
data-driven	1.00	T203A	14	56.2	1.6	3	-		-	neutral
data-driven	0.99	A333L	21	66.5	0.5	3	-		-	inconclusive
data-driven	0.99	Q401A	11	68.1	2.7	3	40.7	0.2	3	stabilising
data-driven	0.98	F288A	35	72.0	3.5	3	41.5	0.7	3	stabilising
data-driven	0.98	E391A	12	63.6	6.0	3	39.4	0.2	3	neutral
data-driven	0.97	G323A	13	74.6	1.8	3	41.5	0.6	2	stabilising

[a] Calculated based on remaining in-gel GFP-signal after single-point temperature challenge thermostability assay.
[b] For n > 1: standard error of the mean (SEM) shown.
[c] Average T_m was calculated from individual T_m estimated for each individual repeat by fitting with a four-parameter dose-response curve (variable slope) by non-linear least-squares fitting in the python package *scipy.stats*

Table S7. Table of residues excluded from selection in $hPTH_1R$ stabilisation

$hPTH_1R$ Residue	B-W ‡	Location	Role/Effect	Ref*
Y191	1.43	TMH1	affects peptide ligand binding and/or potency in multiple class B GPCRs	1
Y195	1.47	TMH1	affects peptide ligand binding and/or potency in multiple class B GPCRs	1
H223	2.50	TMH2	constitutively active receptor	2
M231	2.58	TMH2	interactions with residue 19 of PTH	3
L232	2.59	TMH2	interactions with residue 19 of PTH	3
R233	2.60	TMH2	interactions with residue 19 of PTH - affect peptide ligand binding and/or potency in multiple class B GPCRs - polar Network conserved amongst GPCRs	1, 3, 4
A234	2.61	TMH2	interactions with residue 19 of PTH	3
V235	2.62	TMH2	interactions with residue 19 of PTH	3
S236	2.63	TMH2	interactions with residue 19 of PTH	3
I237	2.64	TMH2	interactions with residue 19 of PTH	3
F238	2.65	TMH2	interactions with residue 19 of PTH	3
V239	2.66	TMH2	interactions with residue 19 of PTH	3
K240	2.67	TMH2	interactions with residue 19 of PTH - affect peptide ligand binding and/or potency in multiple class B GPCRs	1, 3
D241	2.68	TMH2	affects peptide ligand binding and/or potency in multiple class B GPCRs	1
L244	2.71	TMH2	affects peptide ligand binding and/or potency in multiple class B GPCRs	1
Y245	2.72	TMH2	affects peptide ligand binding and/or potency in multiple class B GPCRs	1
L289	3.37	TMH3	affects peptide ligand binding and/or potency in multiple class B GPCRs	1
N295	3.43	TMH3	polar Network conserved amongst GPCRs	4
K359	5.35	TMH5	affects peptide ligand binding and/or potency in multiple class B GPCRs	1
K360	5.36	TMH5	affects peptide ligand binding and/or potency in multiple class B GPCRs	1
T410	6.42	TMH6	constitutively active receptor	2
M414	6.46	TMH6	may be involved in binding.	5
P415	6.47	TMH6	Cross-linking	6
H420	6.52	TMH6	polar Network conserved amongst GPCRs	4
Y421	6.53	TMH6	affects peptide ligand binding and/or potency in multiple class B GPCRs	1
F424	6.56	TMH6	affects peptide ligand binding and/or potency in multiple class B GPCRs	1
M425	6.57	TMH6	may be involved in binding.	5
W437	7.35	TMH7	ligand discrimination	7
Q440	7.38	TMH7	ligand discrimination	7
M441	7.39	TMH7	Cross-linking	6
M445	7.43	TMH7	affects peptide ligand binding and/or potency in multiple class B GPCRs	1
Q451	7.49	TMH7	polar Network conserved amongst GPCRs	4

‡ B-W; Ballesteros–Weinstein numbering scheme

References: 1; Hollenstein et al. (2013), 2; Schipani et al. (1997), 3; Gensure et al. (2003), 4; Liang et al. (2018), 5; Bisello et al. (1998), 6; Gensure et al. (2001), 7; Clark et al. (1998)
